# Cellular senescence in the innervated niche modulates cancer-associated pain: an emerging therapeutic target?

**DOI:** 10.3389/fimmu.2025.1694567

**Published:** 2025-11-11

**Authors:** Fabrizio Antonangeli, Edoardo Arcuri, Angela Santoni

**Affiliations:** 1 Institute of Molecular Biology and Pathology (IBPM), National Research Council (CNR), Rome, Italy; 2 Istituto di Ricovero e Cura a Carattere Scientifico (IRCCS) Regina Elena Cancer Institute, Istituti Fisioterapici Ospitalieri (IFO), Rome, Italy; 3 Department of Molecular Medicine, Sapienza University of Rome, Laboratory Affiliated to Istituto Pasteur Italia - Fondazione Cenci Bolognetti, Rome, Italy; 4 Istituto di Ricovero e Cura a Carattere Scientifico (IRCCS) Neuromed, Pozzilli, Isernia, Italy

**Keywords:** pain, cancer, senescence, SASP, NK cell, neuropathy, neuroinflammation, senolysis

## Abstract

Crosstalk between cancer cells and the nervous system establishes the so-called “innervated niche”. This component of the tumor microenvironment (TME) influences tumor progression and variably regulates the genesis and maintenance of cancer-related pain. Senescence is a cellular stress response emerging as a hallmark of cancer and aging. Through the inflammatory secretome referred to as the senescence-associated secretory phenotype (SASP), senescent cells execute immunomodulation and tissue remodeling, participating in many physio-pathological processes. As inflammation is a key determinant of the TME as well as of neuropathies, in this review article we try to outline the possible role of senescence in the innervated niche. We argue that senescence can contribute to neuroinflammation, which is nowadays recognized as the initial factor triggering both cancer and non-cancer pain, by boosting local inflammation in the TME. At the same time, senescent cells can become targetable elements of the innervated niche to control cancer pain. We describe how the immune system supports the resolution of pain, and we suggest the possibility of harnessing natural killer (NK) cells, the prototype of innate immunity lymphocytes, for therapeutic approaches aimed at pain relief.

## Introduction

### The innervated niche

Even if the concept of tumor microenvironment (TME) dates back to Virchow’s findings on leukocyte infiltration in solid tumors and Paget’s theory of “seed and soil” for metastatic dissemination in the XIX century, only in the last decades cancer research has moved from a tumor cell-centric view based on oncogenes and tumor suppressor genes to a TME-centric perspective (1). Nowadays, the TME is recognized as a key determinant for cancer initiation, progression, and therapy response. TME, which is represented by the biological network of cancer, stromal, endothelial, and immune cells including extracellular metabolites, can be functionally subdivided into different specialized TMEs, such as immune microenvironment, hypoxic microenvironment, cancer stem cell niche and so on (2). Local interaction between nerves and cancer cells has long been observed and is now emerging as an additional peculiar TME that impinges on tumor progression, giving rise to the notion of “innervated niche” (3–5). The innervated niche has been explored so far in the context of neural-cancer interactions focusing on tumor growth and spreading, and cancer-therapy effects on the nervous system. The implementation of new technologies has integrated in the innervated niche the neuroimmune circuits, also facing the role of immune cells in pain processes. Here, we want to add a further player in this liaison: cellular senescence (see **Box 1**). To this aim, after a brief introduction to the innervated niche, how tumors generate chronic pain will be summarized. Then, senescence of tumor and stromal cells as well as of neurons and glial cells will be discussed considering its inflammatory contribution to neuropathy and thus to cancer-associated pain. Finally, how the immune system participates in the processes of pain promotion and control will be outlined, and, in this frame, a specific role of NK cells in targeting senescent cells and hence in senescence-driven pain attenuation will be proposed. We believe that a better understanding of the senescent drivers of cancer pain will be instrumental in the development of novel approaches in analgesia.

Box 1 **Senescence at a glance.**

Senescence is an alternative response to regulated cell death in case of cellular stress. Senescent cells stop proliferating while remaining viable and metabolically active. They display specific morphological and biochemical traits including cellular flattening and enlargement, intracellular vacuolization, increased lysosomal beta-galactosidase (β-Gal) activity, epigenetic and metabolic reprogramming, release of bioactive molecules and inflammatory factors within a massive secretome called senescence-associated secretory phenotype (SASP) (77). Through the SASP, which is rich in proteases (MMP-1, MMP-3), angiogenic factors (VEGF), and cytokines/chemokines (IL-1α, IL-6, IL-8, CCL2), senescent cells perform tissue remodeling and alert the immune system promoting a reparative microenvironment (88, 202). However, if not promptly removed by the immune system, senescent cells accumulate in neoplastic lesions and aging tissues strongly supporting chronic inflammation (203). The SASP is driven by the transcription factors NF-κB, C/EBPβ, and GATA4 and needs activation of the cGAS/STING pathway (204–206). Both innate and adaptive immunity participate in the immunosurveillance of senescent cells, with a pivotal role of natural killer (NK) cells, the prototype of innate immunity lymphocytes (84, 207, 208). Beneficial immune-mediated elimination of senescent cells by NK cells has been observed in tumors, during the resolution of liver fibrosis after damage, and endometrium decidualization (175, 177, 209–211). Macrophages have been reported to be involved in the clearance of senescent cells during embryogenesis and reproductive processes (212, 213). Antitumor activity of CD4 and CD8 T cells has been shown to be enhanced by senescent cell-mediated priming of dendritic cells, suggesting the high potential of senescence as immunogenic process (214–216).

Tumors can be innervated by sympathetic, parasympathetic, or sensory nerves depending on cancer types (6). Neural-cancer communication is bidirectional and can occur via electrochemical, paracrine, systemic, and cancer therapy-mediated interactions (7). Increase of sympathetic innervation in solid tumors is mostly correlated with cancer progression, while parasympathetic signals have both tumor-suppressing and tumor-promoting properties (8, 9). Tumor-promoting action of sympathetic nerves has been ascribed to the adrenergic signaling, as many cancer cells express both the β1- and β2-adrenergic receptors (ARs), and high-grade tumors show higher levels of β-ARs compared to lower-stage diseases (10–12). Catecholamines sustain survival and proliferation of cancer cells by regulating BCL-2 level and BAD phosphorylation, factors implicated in apoptosis, and cyclin D1 expression, an important regulator of cell cycle progression (13–16). Furthermore, catecholamines promote tumor angiogenesis by stimulating the production of the vascular endothelial growth factor (VEGF) (17, 18). The role of cholinergic signaling from parasympathetic innervation is less defined and opposite effects on tumor progression have been reported (19–21).

Cancer cells actively promote tumor innervation by different mechanisms: i) axonogenesis; ii) neurogenesis; iii) reprogramming; iv) perineural invasion. During axonogenesis, neurotrophins, such as the nerve growth factor (NGF) and brain-derived neurotrophic factor (BDNF), semaphorins (axonal guidance molecules), and ephrinB1-containing exosomes secreted by tumor cells drive neuron morphogenesis causing a local increase in nerve density (22). New neurogenesis can originate from cancer stem cells trans-differentiation or neural progenitor cells recruited from the bloodstream (23–26). Reprogramming toward an adrenergic phenotype has been observed in tumor-associated sensory fibers in head and neck cancer (27, 28). In perineural invasion (PNI), cancer cells grow around and invade nerve fibers spreading into the perinerium space. This process provides a facilitated route for metastases and cancer-related pain (29, 30). Although PNI has variable rates in different tumors, PNI invariably correlates with poor prognosis and low survival (31, 32).

### Tumor inflammatory environment is modulated by the innervated niche

A description of the multifold mechanisms by which the nervous system affects tumor growth and regulates immune response in cancer is behind the scope of the present article and we refer to other publications (33, 34). Here, we briefly describe how local inflammation and immunosuppression in the TME, two hallmarks of cancer (35), are influenced by tumor innervation. This is possible because the majority of immune cells express the β2-AR, and cells of the innate immunity express also the α1 and α2 subtypes (36). For example, catecholamines from the sympathetic nervous system influence the function of NK cells, which are lymphocytes of the innate immune system deeply involved in anti-cancer activity (see **Box 2**). NK cells express the D1-like and D2-like dopamine receptors (DRs), which seem to have opposite effect on cytotoxicity and interferon-γ (IFN-γ) production. The D1- and D5-DRs activate the adenylate cyclase signaling, while the D2-, D3-, and D4-DRs inhibit the adenylate cyclase signaling, thereby enhancing and attenuating the effector functions of NK cells, respectively. Also the ARs belong to the G protein-coupled receptor family. NK cells express the α1-AR, the α2-AR, and high levels of the β2-AR but not the β1-AR. Noradrenaline preferentially activates the α-ARs, while adrenaline is an effective stimulator of the β2-AR. In general, adrenaline and noradrenaline, which rapidly increase during acute stress or exercise, seem to inhibit NK cell cytotoxicity and cytokine production as well as mobilize NK cells into the peripheral blood (37).

Box 2 **NK cells: mechanisms of activation and cytotoxicity**

NK cells are large granular lymphocytes belonging to the family of the innate lymphoid cells. They show cytolytic activity against virus-infected and tumor cells without needing a somatic rearrangement of the activating receptors as instead lymphocytes of the adaptive immunity (T and B cells) require (217). Their activation is based on a balance between inhibitory and activating germline-encoded receptors that recognize MHC class I and class I-like molecules that act as signs of cellular stress in cells experiencing different types of insult. Activating receptors encompass the C-type lectin-like receptor NKG2D, the natural cytotoxic receptors NKp30, NKp44, and NKp46, and the co-receptors DNAM-1 and NKp80. Among the activating receptors, NKG2D and DNAM-1 are of great relevance for the immunosurveillance of senescent cells as their ligands are strongly induced in response to senescence (84). In humans, the ligands of NKG2D are MICA, MICB, and ULBP1-6, while mouse NKG2D ligands include RAE-1 (five different isoforms), MULT-1, and H60 (three different isoforms). The ligands of DNAM-1 are PVR (CD155) and Nectin-2 (CD112). Inhibitory receptors include the C-type lectin-like receptor NKG2A, members of the killer cell immunoglobulin-like receptor (KIR) family in humans, and the immune checkpoints TIGIT, LAG3, TIM3, and PD-1 (191). By the tuning of these receptors, NK cells target cells that appear to be missing self or stressed. Furthermore, NK cells are the principal effector cells performing the antibody-dependent cellular cytotoxicity (ADCC) through the CD16 receptor. NK cells are endowed with cytotoxic and immunomodulatory functions. Once activated, NK cells produce large amounts of CCL5, IFN-γ, TNF-α, and hence orchestrate the immune response of other immune cells. The cytolytic effects are carried out through different mechanisms, such as the expression of the death receptor ligands FASL and TRAIL and the release of cytotoxic granules containing pore-forming perforins and granzymes (serine proteases) (218). Different subsets of NK cells exist with only partially overlapping effector functions. In humans, CD56^dim^ CD16^+^ NK cells (paralleled by CD27^-^ CD11b^+^ in mice) are more cytotoxic than regulatory CD56^bright^ CD16^-^ NK cells (murine CD27^+^ CD11b^-^). Uterine and decidual NK cells contribute to vascular remodeling, embryo implantation and fetal growth during pregnancy (219).

The inflammatory reflex represents a well characterized neuroimmune circuity based on the control that the vagus nerve executes on macrophage-dependent production of tumor necrosis factor-α (TNF-α) (33). Following proinflammatory cytokine stimulation of afferent vagus nerves, vagal efferent fibers trigger adrenergic splenic nerves to release noradrenaline that in turn acts on β2-AR-expressing memory T cells in the white pulp. This way stimulated T lymphocytes produce acetylcholine which has inhibitory effect on activated macrophages expressing the α7-nicotinic acetylcholine receptor with the consequence of reducing TNF-α secretion and thus dampening inflammation (38, 39). In addition to this general mechanism, autonomic innervation directly influences the immune cells in the innervated niche. Catecholamines drive a β2-AR-mediated polarization of tumor-associated macrophages (TAMs) toward a pro-tumorigenic M2 phenotype (40, 41). Signaling from the α2- and β2-ARs reduces maturation and migration of dendritic cells to lymph nodes, impairing T cell priming (42, 43). Moreover, the β2-AR signaling mediates direct immunosuppression on tumor antigen-specific CD8 T cells by reducing their proliferation, IFN-γ production, cytolytic effector functions, and glucose metabolism (44, 45). Accordingly, inhibition of the β2-AR signaling elicits an antitumoral microenvironment characterized by an elevated IFN-γ^+^CD8^+^:Treg ratio and reduced expression of the immune checkpoint PD-1 (46).

More in general, adrenergic innervation of lymphoid organs restrains T cell egression from the lymph nodes and bone marrow through CCR7 and CXCR4, while promotes myeloid-derived suppressor cell (MDSC) expansion via the β2-ARs and myeloid cell maturation via the α-ARs in the spleen (47–49). On the contrary, splenic parasympathetic innervation stimulates memory T cells to produce the anti-inflammatory peptide TFF2, which suppresses MDSC expansion in colorectal cancer (50). Regarding sensory fibers, it has been observed that their stimulation by melanoma tumor cells induces the expression of proinflammatory cytokines, such as CCL2, CCL3, CCL5, which speed up MDSC recruitment and tumor growth (51).

Within TME, inflammatory cytokines, in particular IL-6 and IL-8 (CXCL8), contribute to different tumor-promoting mechanisms, such as cancer cell plasticity, angiogenesis, and immunosuppression (52, 53). High levels of IL-6 and IL-8 in the innervated niche are generated upon the engagement of the β-ARs on tumor and immune cells by both noradrenaline from local sympathetic nerves and adrenaline from the blood (54).

## Cancer-associated pain

Pain is a harmful sign and debilitating symptom of advanced cancer. Nociception (the physiological process of perceiving pain) starts through the activation of peripheral pain receptors (nociceptors) represented by the median diameter myelinated Aδ-fibers and small diameter unmyelinated C-fibers, whose cell bodies are located in the dorsal root ganglia (DRG) and trigeminal ganglion (55). From an evolutionary point of view, acute pain has arisen to prevent and protect from tissue damage. Conversely, pain persistence when the original cause is exhausted (chronic pain) or without a detectable cause (sine materia) represents a pathological response (maladaptive) with no protective purpose, resulting from the shift of peripheral neuroinflammation into central neuroinflammation (pain centralization). This response characterized by altered spinal cord and brain neuroplasticity is frequently observed in cancer survivors who have undergone chronic pain triggered by cancer itself or by cancer treatments.

**Figure 1:**
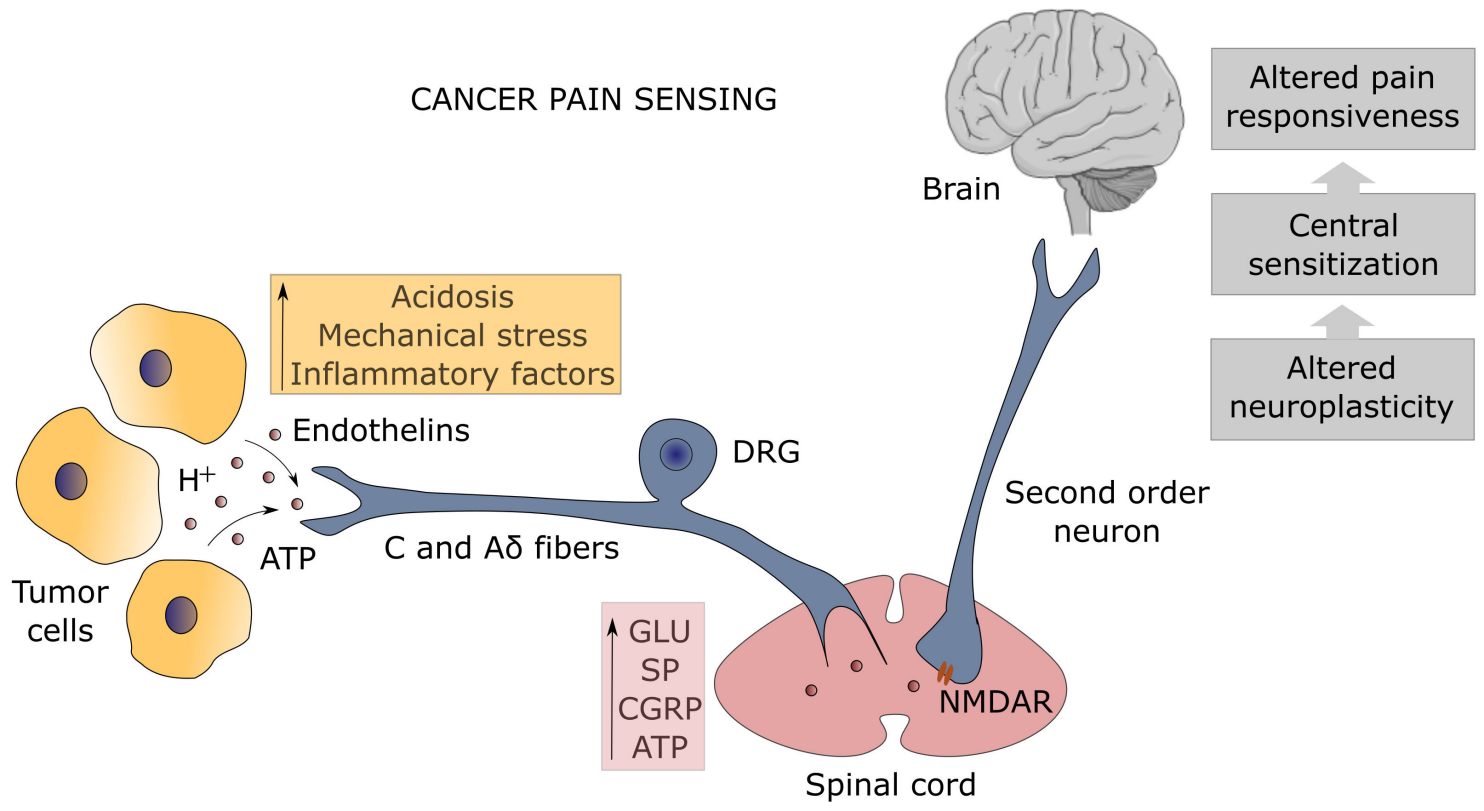
Mechanism of pain sensing at the tumor-nociceptor interface. The unmyelinated C and thinly-myelinated Aδ fibers which represent the primary afferent sensory nerves (known as nociceptors) detect many types of noxious stimuli from tumor cells (orange box). Protons (H+) are sensed by the transient receptor potential vanilloid-1 (TRPV1) channel and the acid-sensing ion channel-3 (ASIC3), adenosine triphosphate (ATP) by the purinergic P_2_X_3_ receptor, endothelins by the endothelin-A receptor, whereas the dorsal-root acid-sensing ion channel (DRASIC) detects the mechanical distension of sensory fibers caused by tumor growth. Activation of nociceptors, whose cell bodies lie in the dorsal root ganglia (DRG), results in the release of neurotransmitters (pink box), such as glutamate (Glu), substance P (SP), calcitonin gene-related peptide (CGRP), and ATP, which transmit the painful signal in the spinal cord to the second order neurons expressing the N-methyl-D-aspartate receptor (NMDAR), leading to spinal cord and brain sensitization with altered pain responsiveness (gray boxes).

Several mechanisms related to cancer contribute to the generation of persistent pain: i) mechanical injury to peripheral nerves induced by tumor growth (56); ii) tumor-mediated tissue acidosis (57); iii) proteolytic activity by tumor cells which leads to neuroactive peptides or direct injury to sensory and sympathetic fibers (58); iv) direct effects of factors released by cancer and stromal cells on nociceptors innervating the tumor-bearing organ (**Figure 1**) (59, 60). Furthermore, cancer-derived inflammation in the innervated niche strongly sensitizes nociceptive nerves leading to allodynia (pain from normally innocuous stimuli) and hyperalgesia (exaggerated response to stimuli of poor intensity) by lowering the action potential threshold or elevating the firing frequency (55).

Inflammation is the pathophysiological response of stromal, vascular, nervous, and immune cells to pathogens and tissue damage aimed at removing the noxious stimulus, promoting the healing process, and restoring tissue integrity (61). Many mediators of inflammation are known to impact on nociceptors enhancing their excitability: histamine, bradykinin, leukotrienes, and prostaglandins from mast cells; adenosine, ATP, and protons from damaged tissues; IL-1β, IL-6, TNF-α, and NGF from macrophages (62); endothelin-1 and NGF from cancer cells. These factors act directly on nociceptors by binding to specific cell surface receptors, leading also to increased sensitivity to temperature and touch (55). For instance, endothelins are detected by the endothelin-A receptor, while ATP binds to the purinergic P_2_X_3_ receptor. Activation of these receptors, as well as the sensing of the mechanical distension of sensory fibers caused by tumor growth detected by the dorsal-root acid-sensing ion channel (DRASIC), lowers the threshold of nociceptor excitability by inducing the phosphorylation of the 1.8 and/or 1.9 sodium channels (Na^+^ channels) (58). Chemokines are other important mediators of cancer-associated pain by recruiting immune cells (63).

Prolonged activation of peripheral fibers contributes to central sensitization through the continuous release of glutamate, substance P (SP), calcitonin-gene related peptide (CGRP), and ATP into the synaptic space, which increases the responsiveness of second order neurons expressing the N-methyl-D-aspartate receptor (NMDAR) in the spinal cord to painful stimuli (**Figure 1**). Central sensitization can also derive from neuroinflammation mediated by glial cell activation, or from the loss of physiological inhibition by inhibitory neurons secreting GABA and glycine, which can lead to perceive pain from non-nociceptive myelinated Aβ primary afferent fibers after innocuous mechanosensitive stimuli (mechanical allodynia) (64). Microglia cells play a pivotal role in speeding up neuroinflammation and pain centralization by triggering astrocyte activation that sensitizes first- and second-order neurons through the release of inflammatory mediators such as TNF-α (65). It should be noted that immune cells, in the effort of a homeostatic mechanism, upon corticotrophin-releasing hormone and noradrenaline stimulation can release β-endorphins which are able to attenuate pain through the engagement of the opioid receptors on sensory nerves (66). This immuno-mediated peripheral analgesia occurs only in the event of an inflammatory response, linking inflammation to both pain-gain and pain-resolution after tissue injury (65, 67). The relationship between opioids and analgesia is ambiguous as opioid-mediated neuroinflammation has emerged. Indeed, morphine and other opioids used for the attenuation of cancer-associated pain can bind the Toll-like receptor 4 accessory protein MD-2 on both microglia and astrocytes eliciting the release of nitric oxide (NO) and production of inflammatory cytokines (68–70). This discovery accounts for the paradoxical consequences of long-lasting opioid treatment, the opioid-induced hyperalgesia (OIH), and marks neuroinflammation as the pathological and pharmacological driving mechanism of chronic pain (71–73).

NGF, besides its role in neuronal development and consequently in the formation of the innervated niche as previously described, is involved in inflammatory hyperalgesia and cancer-associated pain. NGF binds to the neurotrophic high-affinity tyrosine kinase receptor TrkA and the low-affinity receptor p75 expressed on sensory nerves modulating the expression and function of neurotransmitters (SP and CGRP), receptors (bradykinin R), and channels (P2X3, TRPV1, ASIC3 and sodium channels) (60). The transient receptor potential vanilloid-1 (TRPV1) channel is a key component of the pain sensing system being activated by different stimuli including heat, acid, and protons. TRPV1 and the acid-sensing ion channels (ASICs) are responsible for the generation of pain in the acidic milieu that characterizes the TME and the persistent pain occurring in the bone metastases due to the massive tissue acidosis operated by osteoclastic activity (74).

Cancer patients often face chemotherapeutic treatments and several antitumor drugs, including taxanes, the vinca alkaloids, and platinum-based compounds, can induce pain and/or sensory neuropathy, the so-called chemotherapy-induced peripheral neuropathy (CIPN). The mechanisms are poorly understood as these agents have been primarily selected to target dividing cells, but in the case of microtubule-affecting drugs it is reasonable that they impair axonal transport of nerves. Increasing findings also suggest a link between CIPN and a bioenergetic imbalance in sensory neurons caused by drug-induced mitochondrial dysfunction (75). Drug-mediated injury to C and Aδ sensory fibers can lead to myalgia, tingling, cold allodynia, and burning pain in the fingers, whereas damage to Aα and Aβ fibers can result in paresthesias and dysesthesias (60).

## Senescence and cancer pain

### Senescence of tumor and stromal cells

Senescence is a complex cellular program characterized by halted cell cycle and the production of a massive inflammatory secretome called SASP (76). Senescence is triggered by a variety of exogenous and endogenous stressful stimuli including telomere shortening, DNA damage by genotoxic drug, and oxidative stress (77). Cancer cells can undergo senescence due to oncogenic proliferative stress (the so-called oncogene-induced senescence or OIS) or therapy-induced insult (the the so-called therapy-induced senescence or TIS). Senescent tumor cells have both tumor-suppressing and tumor-promoting properties, depending on the context (premalignant lesion or neoplastic tissue) and TME (cold versus hot tumors) (78, 79). A further layer of complexity is provided by the induction of senescence in stromal cells (80–83). Senescent cells are in close connection with the immune system as the SASP drives the recruitment and activation of immune cells and, in turn, immune cells recognize and target senescent cells (84, 85). Consequently, senescence deeply modifies cancer immune landscape (86). In addition, SASP factors impact on tissue homeostasis performing tissue remodeling and repair (87–89).

Cancer cells and cells of the immune system have a continuous dialog conceptualized in the cancer immunoediting theory (90). In this scenario, senescence affects the three phases of cancer immunoediting, i.e. elimination, equilibrium, escape. For a comprehensive review on the topic see (91). Here we want to highlight how senescent cells within the innervated niche can influence cancer-associated pain.

SASP composition is extremely heterogeneous and dynamic, depending on cell type and cause of senescence (92–94). Nevertheless, some factors are shared among the conditions and are discussed below regarding their capacity to affect cancer-evoked pain (**Table 1**).

**Table 1:** SASP factors for which a cascade to enhanced nociception is known.

SASP factor	Target cell/pathway	Downstream target	Reference
G-CSF, GM-CSF	Nerve/JAK-STAT3	↑NaV1.8, ↑TRPV1, ↑CGRP	(95)
IL-6	DRG/JAK-PI3K	↑TRPV1	(98, 99)
IL-1β	Spinal cord	↑NMDAR	(119)
M-CSF	Macrophage	↑TRPA1	(142, 143)

**Figure 2:**
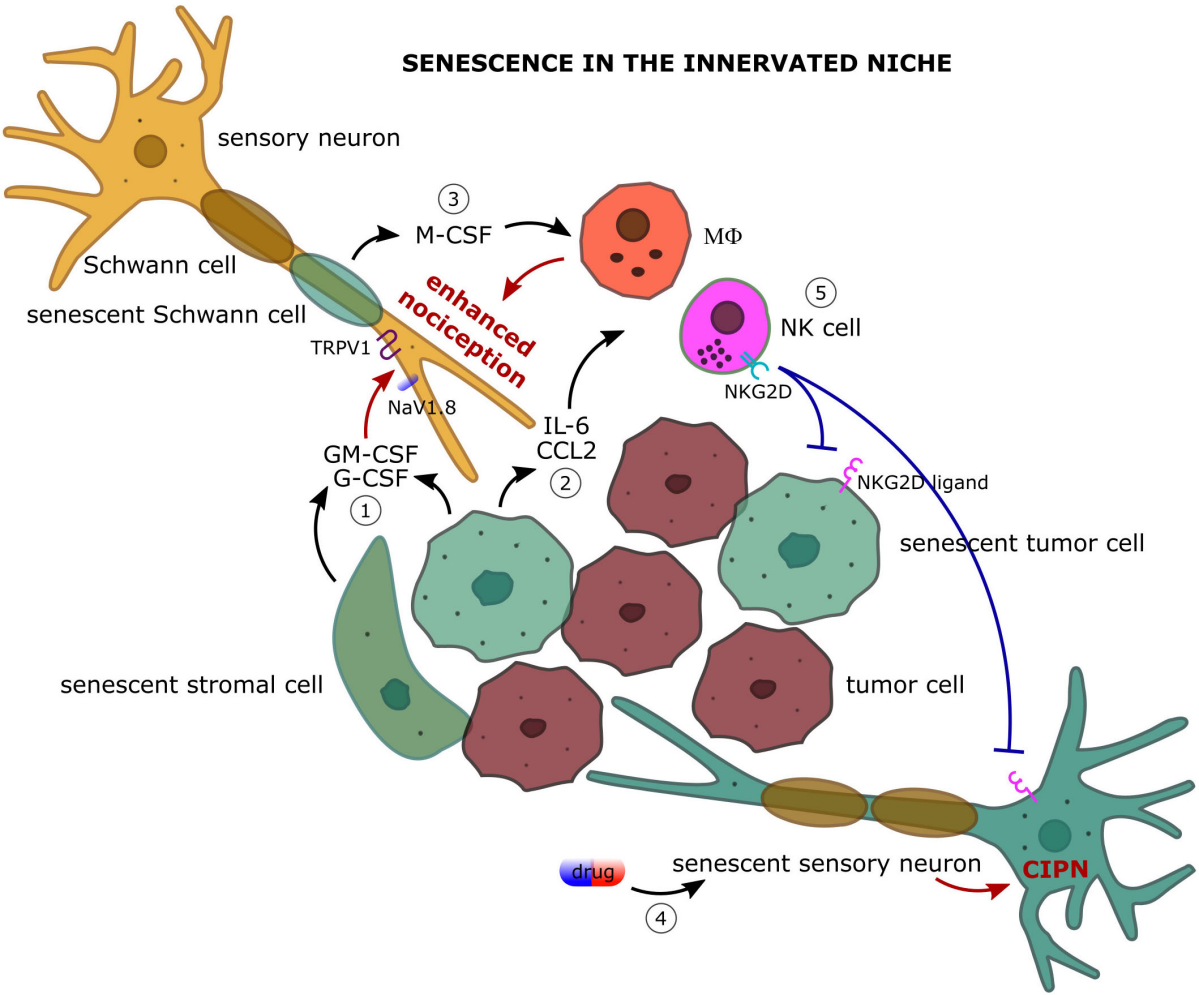
The complex dialog among the actors of the innervated niche: focus on how cellular senescence impacts on cancer-associated pain. 1) Senescent tumor and stromal cells secrete large amount of granulocyte colony stimulating factor (G-CSF) and granulocyte-macrophage colony stimulating factor (GM-CSF) which upregulate the expression of the sodium channel NaV1.8 and the heat-activated channel TRPV1 on primary afferent nociceptors leading to enhanced pain perception upon nociceptive stimulation. 2) Senescent cells are a robust source of inflammatory cytokines (e.g. IL-6) and chemokines (e.g. CCL2) and actively recruit immune cells, such as macrophages (MΦ) and natural killer (NK) cells, in the innervated niche. Macrophage-promoted inflammation and oxidative stress strongly sensitize nociceptive nerves leading to enhanced nociception. 3) Senescent Schwann cells contribute to the recruitment and proliferation of the macrophage population in the innervated niche by producing the macrophage colony stimulating factor (M-CSF). 4) Senescence of sensory neurons following chemotherapy can participate in the pathogenesis of chemotherapy-induced peripheral neuropathy (CIPN). 5) Senescence-attracted NK cells target both tumoral and neuronal senescent cells through NKG2D/NKG2D ligand interaction providing an immune cell-mediated mechanism of pain control.

*Hematopoietic colony stimulating factors*. Signaling generated from the granulocyte colony stimulating factor (G-CSF) and granulocyte-macrophage colony stimulating factor (GM-CSF) has been linked to pancreatic adenocarcinoma and bone cancer pain. Starting from the finding that receptors for both cytokines are expressed on pancreatic nerves in biopsy from healthy individuals and individuals with pancreatic tumors showing hypertrophic nerves, the signal transduction investigated in a mouse sarcoma model of bone tumor–induced pain has been shown to be mediated by the JAK-STAT3 pathway and to lead to the upregulation of the sodium channel NaV1.8 and the heat-activated channel TRPV1 (95). This nociceptor sensitization is accompanied by an increased release of the pain-related peptide CGRP upon nociceptive stimulation. In this way, G-CSF and GM-CSF are responsible for thermal and mechanical hyperalgesia in bone metastases (**Figure 2**). It should be noted that G-CSF and GM-CSF in the TME also contribute to the formation of the innervated niche by promoting both cancer cell proliferation and the branching of tumor innervating fibers (95). As G-CSF and GM-CSF are two factors of the SASP, it is expected that senescence in the TME can strongly enhance pain perception by increasing the local level of G-CSF and GM-CSF. Even if the actual levels have been reported to be regulated by the states of senescence, namely p53 status (functional or mutated), both senescent tumor cells and senescent stromal cells, such as fibroblasts, produce high amount of GM-CSF and, in some cases, of G-CSF (96, 97).

*IL-6.* IL-6 is a well-known SASP factor involved in autocrine and paracrine senescence. It regulates immune response and drives somatic cell reprogramming. Being a potent inflammatory cytokine, in the TME it is associated with tumorigenesis by promoting cell proliferation, migration, metastasis, angiogenesis, and immune evasion (88). Regarding pain experience, high levels of IL-6 have been observed in the DRG and spinal cord of different rat models of pathological pain where an IL-6/JAK/PI3K/TRPV1 signaling cascade has been characterized (98, 99). Administration of IL-6 provokes mechanical allodynia and thermal hyperalgesia (100). Accordingly, IL-6 knockout mice show reduced mechano-allodynia following spinal nerve lesion (101). IL-6 mediates nociceptive plasticity in part by enhancing protein translation in sensory neurons (102, 103). Patients with painful peripheral neuropathy have been shown to have elevated local levels of IL-6 and IL-8 in the affected skin (104). IL-6 has also been implicated in CIPN, even if with conflicting findings. A protective role has been reported in three animal models of paclitaxel, cisplatin and vincristine-induced neuropathies, while a reduced incidence of vincristine-induced mechanical allodynia has been found in IL-6 knockout mice (105, 106). It should be noted that different animals were used (rat versus mouse), possibly accounting for the discrepancies. Supporting a positive correlation, two clinical studies, one in women with breast cancer after chemotherapy (taxanes) and one in patients with metastatic prostate cancer who received chemotherapy (docetaxel), point to an association of high plasma levels of IL-6 and soluble IL-6 receptor with CIPN intensity (107, 108). Nevertheless, caution should be taken before drawing conclusions as different bias could affect studies involving cancer patients experiencing chronic pain. IL-6 is also involved in the processes of inflammaging, and it is now well recognized that senescent cells that accumulate in aged tissues are great producers of systemic IL-6 (109, 110). A similar role can be assumed for senescent cells in the TME. It is reasonable that senescent cell-derived IL-6 can contribute both systemically and locally to the establishment of chronic inflammation paving the way to persistent pain. However, this scenario still needs to be experimentally validated.

*CCL2*. CCL2 is the most representative chemokine of the SASP involved in the recruitment of monocytes/macrophages, MDSCs, and NK cells (78, 111). It has been reported that CCL2 can be produced by neurons of the DRG in rodents and that mice lacking the chemokine receptor CCR2 abrogate the development of mechanical allodynia, suggesting that CCL2 can contribute to pain generation by a direct action on neurons (an intracellular Ca^2+^ signaling has been observed in DRG cells treated with CCL2) or by indirect inflammatory effects mediated by the immune system (112–114). In this context, senescent tumor cells can be a robust source of CCL2 and possibly participate in neuropathic pain (**Figure 2**).

*IL-1*. IL-1α and IL-1β are highly produced within the SASP, driving a critical function in the establishment of the senescent phenotype (115–117). A role in pain generation can be inferred as usage of the IL-1R antagonist anakinra has been reported to reduce mechanical hyperalgesia in rat models of bone cancer pain by dampening the NMDAR signaling and the PI3K-mTOR pathway in the spinal cord and brain, respectively (118, 119).

*Bioactive lipids*. There is scarce information about biologically active lipids in the SASP. Production of leukotrienes has been documented in senescent fibroblasts in correlation with lung fibrosis (120). Senescent dermal and prostatic fibroblasts have been reported to secrete prostaglandin E_2_ due to cyclooxygenase-2 upregulation during senescence (121). Lipid biosynthetic pathways have been shown to be orchestrated in a time-dependent manner following the induction of senescence and have been suggested to be implicated in the well-known role of senescent cells in wound healing (122, 123). Eicosanoids are important effectors of inflammation, and this may represent a further mechanism through which senescent cancer and stromal cells contribute to neuroinflammation-caused pain in the innervated niche.

### Senescence of neurons and glial cells

Being associated with stable growth arrest, cellular senescence is commonly observed in proliferating cells, but recent evidence suggests that also postmitotic and terminally differentiated cells, such as neurons and glial cells, are able to undertake a senescence program following appropriate stimuli (124, 125). The unfolded protein response (UPR) is of particular importance in postmitotic specialized cells that have limited turnover capacity. Sustained activation of the UPR due to accumulation of stress granules and protein aggregation may account for the establishment of the senescent phenotype in aged neurons (126, 127). Indeed, it has been reported in mouse models of tauopathies and postmortem specimens from brains of patients with Alzheimer’s disease that the affected neurons show a canonical senescence stress response with DNA damage, aberrant cellular respiration, upregulation of cell cycle inhibitors, resistance to cell death, and inflammation mediated by NF-κB (128). Accordingly, treatment with senolytics (dasatinib plus quercetin) in mice has been shown to reduce the senescence signature (128). Senescent neurons share different phenotypic features of senescent mitotic cells, although not all, such as enhanced β-Gal activity, DNA damage, SASP (126). The use of the β-Gal activity, as well as of lipofuscin accumulation, as marker of neuronal senescence deserves particular attention due to the positive staining occurring in normal neurons throughout the lifespan, especially in cerebellar Purkinje neurons, hippocampal CA2 neurons, and a subset of cortical neurons (128, 129). Likewise primary fibroblasts, primary rat hippocampal neurons in long-term cultures display characteristics of senescence (senescence-associated β-Gal activity, p16 accumulation, and loss of lamin B1) after experiencing proteostasis failure (130). DNA damage accumulating in aging neurons is causative of a senescence-like phenotype dependent on p21 (131).

Relevant to our discussion, neuronal senescence has been described in the event of CIPN at least in mouse models. Cisplatin-induced DNA damage in DRG neurons is not associated with apoptotic cell death but with a senescence response, as revealed by lysosomal β-Gal activity and p21 upregulation, accumulation of lipofuscin granules and morphological changes (enlarged endoplasmic reticulum and larger mitochondria), lack of caspase-3 cleavage (132). Remarkably, the clearance of cisplatin-induced senescent DRG neurons by a pharmacological approach with the ABT263 compound (Navitoclax, daily intraperitoneal injections at 50 mg/kg for 2 cycles lasting 5 days with a 16-day rest period between) or genetic deletion of p16^+^ senescent cells (p16-3MR transgenic mouse) improves symptoms of CIPN as assessed by mechanical (von Frey test) and thermal (hot plate test) stimulation at least until three months after 2 cycles of 2.3 mg/kg cisplatin treatment (5 days on-5 days off-5 days on), suggesting that senescent neurons play a role in the pathogenesis of CIPN (133). ABT263 is an inhibitor of the anti-apoptotic proteins BCL-2 and BCL-xL and selectively targets senescent cells, which are known to upregulate anti-apoptotic factors (134). Usage of senolytics is currently under investigation for the treatment of age-associated diseases (135), leading to hypothesize also a possible application in the management of CIPN, considering that CIPN is among the most common dose-limiting adverse effects of anticancer drugs. Based on encouraging results in preclinical models, first- and second-generation senolytics have landed into clinical trials in humans. Only mild to moderate reversible adverse events have been reported so far but the effectiveness of senolytics for the tested pathologies remains scant. Regarding pain, different trials (NCT03513016, NCT04129944, NCT04210986, NCT04229225, NCT04349956, NCT04770064) aimed at targeting senescence to reduce osteoarthritis pain by using nutlin-3a (UBX0101) or fisetin are still ongoing or failed to achieve the primary endpoint of improving pain in patients with osteoarthritis of the knee (135, 136). Interestingly, a role for cellular senescence in long-term pain has been postulated to justify the male-specific sex-biased chronic pain observed in a mouse model of nerve injury, where accumulation of senescent cells in the spinal cord due to telomere shortening has been reported only in male mice (137).

Schwann cells are the glial cells of the peripheral nervous system designed to the myelination of nerves. Schwann cells not only are involved in the saltatory nerve conduction but also contribute to nerve regeneration after injury and participate in cancer-evoked pain (138). In homeostatic conditions Schwann cells uphold pain relief by protecting neurons and counteracting demyelination whereas during inflammation they secrete a variety of neurotrophic factors (NGF and BDNF) that guide axon repair but also exacerbate pain (139). In response to nerve injury, Schwann cells assume a non-myelinating phenotype with proliferation capacity aimed at regulating the Wallerian degeneration of axon and subsequent regeneration. The efficiency of this process has been linked in mice to the duration of neuropathic pain, making Schwann cells a promising target for the management of chronic pain (140, 141). Schwann cells are also involved in a pain-eliciting circuity with macrophages, demonstrated so far only mice. Macrophages display clear pro-algesic effects at the site of nerve injury by feedforwarding oxidative stress. In the context of cancer, the high levels of reactive oxygen species (ROS) that characterized the TME trigger the transient receptor potential ankyrin 1 (TRPA1) on Schwann cells that in turn release M-CSF promoting the recruitment and expansion of the macrophage population which, in a positive feedback, increases oxidative stress and overstimulates the sensory neurons thus sustaining allodynia and spontaneous pain (**Figure 2**) (142, 143). Additionally, Schwann cells have been reported to reciprocally interact with cells of oral squamous cell carcinoma in both mice and humans via adenosine and TNF-α, with the result of increasing the pro-nociceptive mediators IL-6 and NGF (144, 145). Considering that this role of Schwann cells in the modulation of cancer-associated pain has been correlated, at least in some circumstances, to the production of chemokines (M-CSF, TNF-α, and IL-6) which are abundant in the SASP of senescent cells, the discovery that Schwann cells can undergo senescence discloses further conceivable mechanisms of pain generation and, at the same time, new possible opportunities for the management of cancer-evoked pain (146–148).

### Role of the immune system: the cogent case of NK cells

The immune system is deeply involved in the processes of pain generation and control [for a review see (149)]. As previously outlined, immune cell-mediated neuroinflammation triggers long-term pain by sensitization of sensory fibers. Moreover, immune cell-executed cytotoxic effects directly damage nerves. On the other hand, the immune system participates in several ways to the resolution of pain (150). This seemingly contradictory role is consistent with the functions of the immune system aimed at tissue healing after removal of harmful stimuli. This goal is achieved through the plasticity of immune cells which physiologically occurs during the healing processes and can be exploited for therapeutic interventions. Regarding pain attenuation, immune cells promote analgesia by secreting both anti-inflammatory cytokines (for instance IL-10 hinders the production of TNF-α) and pro-reparative cytokines (such as IL-4) (151). IL-10 has been shown to counteract mechanical hypersensitivity after CIPN in DRG neurons treated with cisplatin (152). IL-4-mediated effects are believed to be due, at least in part, to the analgesic properties of opioids produced by M2-polarized macrophages (153). Endogenous opioid peptides and lipidic endocannabinoids supplied by immune cells, e.g. macrophages, T cells, and microglia, are other important modulators of pain (151).

Among the effector functions of immune cells in the innervated niche, cytotoxicity is critical to target cancer cells but also can lead to painful nerve injury (154). At the same time, it is now appreciated that cytotoxic immune cells, especially macrophages, neutrophils, and NK cells, contribute to the neuropathic pain resolution by clearing damaged neurons and performing phagocytic removal of debris (155). A transient inflammatory wave driven by the activation of neutrophils, macrophages, and mast cells has been associated with musculoskeletal pain resolution in humans, preventing the transition from acute to chronic pain. Accordingly, inhibition of the inflammatory response by steroids in mice has been shown to induce analgesia in the short term but to delay full recovery from pain in the long run (156).

NK cells are lymphocytes of the innate immune system with pronounced cytotoxic and immunomodulatory functions. Involvement of NK cells in the processes of neuropathic pain is proved by different lines of evidence. Activation of NK cells has been observed in both humans and mice after acute painful stimuli, electric and heat shock, respectively (157, 158). An inverse correlation between NK cell frequency in the cerebrospinal fluid and mechanical pain sensitivity has been reported in patients with neuralgia, hypothesizing a role of NK cells in preventing central sensitization (159). The analgesic effect of electroacupuncture has been correlated with the cytotoxic activity of splenic NK cells, at least in rat models of pain (160, 161).

Injury to peripheral nerve is followed by the fragmentation of the damaged axons by a neuron-intrinsic mechanism (cytoskeletal destabilization) called Wallerian degeneration that leads to the elimination of the nerve stump distal to the site of injury but preserves the cell body (162). A permissive milieu for axonal regeneration is then promoted by debris clearance and glial reactivation. First evidence of an NK cell contribution to neuronal degeneration derived from studies of mononuclear inflammatory cell infiltration in athymic nude rats, which lack T lymphocytes, after exposure to guanethidine, an adrenergic blocking agent causing the death of sympathetic neurons resident in the superior cervical ganglia (163, 164). Furthermore, DRG neurons can be killed directly by syngenic IL-2-activated NK cells, but the efficiency of the process is strictly dependent on the lack of glia cells, restricting the NK-mediated mechanism of elimination to damaged neurons (165). The seminal work by Davies and colleagues has further demonstrated that in the context of peripheral nerve injury in adult mice NK cells complement the Wallerian degeneration by targeting damaged sensory nerves, thus participating in peripheral nerve regeneration (166). In particular, they showed that the injured neurons flag themselves as damaged by expressing the NKG2D ligand RAE-1 to trigger NK cell cytotoxicity. NK cells extravasate and infiltrate the nerves by few days from injury and promote axon degeneration through granzyme-B. RAE-1 protein is anatomically restricted to the peripheral axons of injured sensory neurons either by anterogradely transport along the axon or by mRNA local translation, indicating that NK cell-neuron cytotoxic interaction occurs at the peripheral site saving the cell body. Strikingly, NK cell activity is accompanied by reduced hypersensitivity to mechanical stimulation, a surrogate marker of chronic neuropathic pain, providing a neuron-extrinsic immune cell-mediated mechanism of pain control. Abolishing NK cell activity by anti-NK cell antibody leads to reduced degenerating fibers but more remaining abnormal fibers, as assessed by myelin and axoplasm integrity, which likely conduct the painful sensory response. Indeed, it is reasonable that an efficient clearance of the injured fibers is required to avoid the aberrant sensing of pain that characterizes damaged but functionally active sensory axons or mistargeted re-innervating neurons (167, 168). It is tempting to speculate that NK cells could work as “cellular microsurgeons” to pruning the mis-wired endings of sensory nerve (169). Genetic and chemical approaches to target nociceptors and cope with acute and chronic pain have been already proposed in clinical veterinary and human pain states (e.g. with resiniferatoxin to target TRPV1-expressing small-diameter sensory neurons) (170–172).

Senescent cells are a preferential target of NK cells (84). Not only senescent cells actively recruit NK cells by secreting a plethora of chemokines (CCL2, CCL4, CCL5, CXCL10, CX3CL1), but also sustain and trigger NK cell activity through cytokine production (IL-15, IL-18, TNF-α) and by expressing on the cell surface the ligands of the NK cell activating receptors (111, 173–177). The stress-induced ligands of the receptors NKG2D and DNAM-1 are strongly up-regulated by tumor cells following OIS and TIS and senescent cells are targeted by NK cells through cytotoxic granule exocytosis and not death receptor signaling (178, 179). This mechanism promotes the immune surveillance of senescent cells in different physio-pathological settings and may be relevant also for the resolution of the senescence-driven pain in the innervated niche (**Figure 2**). Supporting this view, the capacity of NK cells to target DRG neurons via RAE-1/NKG2D interaction has been demonstrated at least *in vitro* (180). Transient senescence has pro-regenerative functions, and a senescence signature has been observed after peripheral nerve injury which declines over time, suggesting a reprogramming of the senescent phenotype or an immune-mediated clearance of the induced senescent cells (181). It should be noted that the capacity of senescent cells of attracting NK cells within the TME can also affect the non-senescent cell compartment, as not senescent tumor cells are targeted by NK cells, and activated T cells can become susceptible to autologous NK lysis via NKG2D/NKG2D ligand interaction through granule exocytosis leading to inflammation quenching (182). Senescent cell accumulation and gut dysbiosis are two shared features of aging and cancer (183–185). It has been shown in different mouse models that gut microbiota modulates NK cell effector functions against tumor cells and there is also a similar functional correlation in analyses from humans (186–189). As a speculative hypothesis, adoption of a healthy diet or a diet supplemented with probiotics and prebiotics to enhance NK cell activity could be part of a strategy to target senescent tumor cells and thus promote cancer-associated pain relief. The therapeutic opportunity of NK cells for the treatment of neuropathic pain is not new [see (169)], but the disclosure of the role of senescence in the innervated niche could extend the field of application.

## Future directions

Cancer, nervous and immune systems are deeply interweaved, demanding holistic approaches for the management of cancer-associated pain. As neuropathic pain shows features of chronic neuroinflammation and the TME is characterized by an inflammatory milieu, we are in need to pinpoint the role of cellular senescence in the innervated niche and map the precise source of inflammatory factors to weigh the contribution of senescent cells. Cellular senescence is a powerful driver of inflammation but at the same time is becoming a targetable element offering an innovative line of intervention (135, 190). Approaches currently under investigation for the targeting of senescent cells in cancer and aging diseases could be explored to treat cancer-evoked pain. Senolytic strategies are attractive but still have concerns: i) on- and off-target effects have not been fully addressed; ii) senescent cell markers are not univocal leading to misleading interpretations about senescence burden and more reliable biomarkers are essential to evaluate treatment efficacy; iii) heterogeneity of senescent cells makes difficult to weigh up beneficial and detrimental effects; iv) more studies devoted to treatment regimen and frequency are needed. Along with senolytics, strategies aimed at harnessing the immune system to tackle senescent cells are promising. These include: i) adoptive transfer of boosted NK cells (191); ii) improved NK cell cytotoxicity by targeting the CD94/NKG2A inhibitory receptor using anti-NKG2A therapeutic mAb (i.e. monalizumab) (192, 193); iii) ADCC triggered by anti-senescent cell-specific mAb (i.e. anti-DDP4 mAb) (194); iv) CAR-T cells specific for senescent cells, namely CAR-T cells that recognize the surface senescence-specific marker urokinase-type plasminogen activator receptor (uPAR) or NKG2D-CAR-T cells (195, 196); v) anti-PD-L1 or anti-PD-L2 immune checkpoint inhibitory therapies that enhance the killing capacity of cytotoxic lymphocytes against PD-L1- and PD-L2-expressing senescent cells (197, 198). Among these options, therapies based on anti-NKG2D and anti-PD-L1 monoclonal antibodies are already in clinical use with a favorable safety profile (199, 200). Compared to CAR-T cells, NK cells are emerging as a valid alternative with a safer profile, opening the possibility of an “off-the shelf” therapy (201). Senescence has undoubted immune-stimulating features, but as there is now evidence that senescence can arise not only in tumor cells but also in neurons and glial cells, due to cellular stress conditions or because of anticancer therapy-mediated effects, NK cell-based therapies against senescent cells should be carefully calibrated to the right targets to avoid unwanted and unpredictable side effects. For example, NK cell-killing of senescent neuroblasts in the dentate gyrus of hippocampus has been associated to impaired neurogenesis and loss of cognitive functions. Immunotherapies have revolutionized cancer treatment, disclosing the inherent power of the immune system plasticity. This finding should encourage the exploration of immune-mediated analgesia, a new burgeoning field across cancer neuroscience and algology.

## References

[1] Maman S Witz IP . A history of exploring cancer in context. Nat Rev Cancer. (2018) 18:359–76. doi: 10.1038/s41568-018-0006-7, PMID: 29700396

[2] Zhou Y Cheng L Liu L Li X . Nk cells are never alone: crosstalk and communication in tumour microenvironments. Mol Cancer. (2023) 22:34. doi: 10.1186/s12943-023-01737-7, PMID: 36797782 PMC9933398

[3] Jin MZ Jin WL . The updated landscape of tumor microenvironment and drug repurposing. Signal Transduct Target Ther. (2020) 5:166. doi: 10.1038/s41392-020-00280-x, PMID: 32843638 PMC7447642

[4] Shi J Xu J Li Y Li B Ming H Nice EC . Drug repurposing in cancer neuroscience: from the viewpoint of the autophagy-mediated innervated niche. Front Pharmacol. (2022) 13:990665. doi: 10.3389/fphar.2022.990665, PMID: 36105204 PMC9464986

[5] Young HH . On the presence of nerves in tumors and of other structures in them as revealed by a modification of ehrlich’s method of “Vital staining” with methylene blue. J Exp Med. (1897) 2:1–12. doi: 10.1084/jem.2.1.1, PMID: 19866822 PMC2117917

[6] Reavis HD Chen HI Drapkin R . Tumor innervation: cancer has some nerve. Trends Cancer. (2020) 6:1059–67. doi: 10.1016/j.trecan.2020.07.005, PMID: 32807693 PMC7688507

[7] Monje M Borniger JC D’Silva NJ Deneen B Dirks PB Fattahi F . Roadmap for the emerging field of cancer neuroscience. Cell. (2020) 181:219–22. doi: 10.1016/j.cell.2020.03.034, PMID: 32302564 PMC7286095

[8] Hernandez S Serrano AG Solis Soto LM . The role of nerve fibers in the tumor immune microenvironment of solid tumors. Adv Biol (Weinh). (2022) 6:e2200046. doi: 10.1002/adbi.202200046, PMID: 35751462

[9] Silverman DA Martinez VK Dougherty PM Myers JN Calin GA Amit M . Cancer-associated neurogenesis and nerve-cancer cross-talk. Cancer Res. (2021) 81:1431–40. doi: 10.1158/0008-5472.CAN-20-2793, PMID: 33334813 PMC7969424

[10] Eng JW Kokolus KM Reed CB Hylander BL Ma WW Repasky EA . A nervous tumor microenvironment: the impact of adrenergic stress on cancer cells, immunosuppression, and immunotherapeutic response. Cancer Immunol Immunother. (2014) 63:1115–28. doi: 10.1007/s00262-014-1617-9, PMID: 25307152 PMC4325998

[11] Moretti S Massi D Farini V Baroni G Parri M Innocenti S . Beta-adrenoceptors are upregulated in human melanoma and their activation releases pro-tumorigenic cytokines and metalloproteases in melanoma cell lines. Lab Invest. (2013) 93:279–90. doi: 10.1038/labinvest.2012.175, PMID: 23318885

[12] Sardi I Giunti L Bresci C Buccoliero AM Degl’innocenti D Cardellicchio S . Expression of beta-adrenergic receptors in pediatric Malignant brain tumors. Oncol Lett. (2013) 5:221–5. doi: 10.3892/ol.2012.989, PMID: 23255924 PMC3525361

[13] Sastry KS Karpova Y Prokopovich S Smith AJ Essau B Gersappe A . Epinephrine protects cancer cells from apoptosis via activation of camp-dependent protein kinase and bad phosphorylation. J Biol Chem. (2007) 282:14094–100. doi: 10.1074/jbc.M611370200, PMID: 17353197

[14] Shan T Ma Q Zhang D Guo K Liu H Wang F . Beta2-adrenoceptor blocker synergizes with gemcitabine to inhibit the proliferation of pancreatic cancer cells via apoptosis induction. Eur J Pharmacol. (2011) 665:1–7. doi: 10.1016/j.ejphar.2011.04.055, PMID: 21570961

[15] Zhang D Ma Q Wang Z Zhang M Guo K Wang F . Beta2-adrenoceptor blockage induces G1/S phase arrest and apoptosis in pancreatic cancer cells via ras/akt/nfkappab pathway. Mol Cancer. (2011) 10:146. doi: 10.1186/1476-4598-10-146, PMID: 22118662 PMC3250953

[16] Zhang D Ma QY Hu HT Zhang M . Beta2-adrenergic antagonists suppress pancreatic cancer cell invasion by inhibiting creb, nfkappab and ap-1. Cancer Biol Ther. (2010) 10:19–29. doi: 10.4161/cbt.10.1.11944, PMID: 20424515

[17] Chakroborty D Sarkar C Basu B Dasgupta PS Basu S . Catecholamines regulate tumor angiogenesis. Cancer Res. (2009) 69:3727–30. doi: 10.1158/0008-5472.CAN-08-4289, PMID: 19383906 PMC7880556

[18] Kuol N Stojanovska L Apostolopoulos V Nurgali K . Role of the nervous system in tumor angiogenesis. Cancer Microenviron. (2018) 11:1–11. doi: 10.1007/s12307-018-0207-3, PMID: 29502307 PMC6008269

[19] Bautista M Krishnan A . The autonomic regulation of tumor growth and the missing links. Front Oncol. (2020) 10:744. doi: 10.3389/fonc.2020.00744, PMID: 32477953 PMC7237572

[20] Renz BW Tanaka T Sunagawa M Takahashi R Jiang Z Macchini M . Cholinergic signaling via muscarinic receptors directly and indirectly suppresses pancreatic tumorigenesis and cancer stemness. Cancer Discov. (2018) 8:1458–73. doi: 10.1158/2159-8290.CD-18-0046, PMID: 30185628 PMC6214763

[21] Tibensky M Mravec B . Role of the parasympathetic nervous system in cancer initiation and progression. Clin Transl Oncol. (2021) 23:669–81. doi: 10.1007/s12094-020-02465-w, PMID: 32770391

[22] Vermeer PD . Exosomal induction of tumor innervation. Cancer Res. (2019) 79:3529–35. doi: 10.1158/0008-5472.CAN-18-3995, PMID: 31088834 PMC6635078

[23] Ayala GE Dai H Powell M Li R Ding Y Wheeler TM . Cancer-related axonogenesis and neurogenesis in prostate cancer. Clin Cancer Res. (2008) 14:7593–603. doi: 10.1158/1078-0432.CCR-08-1164, PMID: 19047084

[24] Dyachuk V Furlan A Shahidi MK Giovenco M Kaukua N Konstantinidou C . Neurodevelopment. Parasympathetic neurons originate from nerve-associated peripheral glial progenitors. Science. (2014) 345:82–7. doi: 10.1126/science.1253281, PMID: 24925909

[25] Lu R Fan C Shangguan W Liu Y Li Y Shang Y . Neurons generated from carcinoma stem cells support cancer progression. Signal Transduct Target Ther. (2017) 2:16036. doi: 10.1038/sigtrans.2016.36, PMID: 29263908 PMC5657421

[26] Mauffrey P Tchitchek N Barroca V Bemelmans AP Firlej V Allory Y . Progenitors from the central nervous system drive neurogenesis in cancer. Nature. (2019) 569:672–8. doi: 10.1038/s41586-019-1219-y, PMID: 31092925

[27] Amit M Takahashi H Dragomir MP Lindemann A Gleber-Netto FO Pickering CR . Loss of P53 drives neuron reprogramming in head and neck cancer. Nature. (2020) 578:449–54. doi: 10.1038/s41586-020-1996-3, PMID: 32051587 PMC9723538

[28] Hunt PJ Amit M . Head and neck cancer exosomes drive microrna-mediated reprogramming of local neurons. Extracell Vesicles Circ Nucl Acids. (2020) 1:57–62. doi: 10.20517/evcna.2020.04, PMID: 33554224 PMC7861575

[29] Marchesi F Piemonti L Mantovani A Allavena P . Molecular mechanisms of perineural invasion, a forgotten pathway of dissemination and metastasis. Cytokine Growth Factor Rev. (2010) 21:77–82. doi: 10.1016/j.cytogfr.2009.11.001, PMID: 20060768

[30] Seefeld PH Bargen JA . The spread of carcinoma of the rectum: invasion of lymphatics, veins and nerves. Ann Surg. (1943) 118:76–90. doi: 10.1097/00000658-194311810-00005, PMID: 17858252 PMC1617674

[31] Bapat AA Hostetter G Von Hoff DD Han H . Perineural invasion and associated pain in pancreatic cancer. Nat Rev Cancer. (2011) 11:695–707. doi: 10.1038/nrc3131, PMID: 21941281

[32] Liebig C Ayala G Wilks JA Berger DH Albo D . Perineural invasion in cancer: A review of the literature. Cancer. (2009) 115:3379–91. doi: 10.1002/cncr.24396, PMID: 19484787

[33] Dantzer R . Neuroimmune interactions: from the brain to the immune system and vice versa. Physiol Rev. (2018) 98:477–504. doi: 10.1152/physrev.00039.2016, PMID: 29351513 PMC5866360

[34] Wang W Li L Chen N Niu C Li Z Hu J . Nerves in the tumor microenvironment: origin and effects. Front Cell Dev Biol. (2020) 8:601738. doi: 10.3389/fcell.2020.601738, PMID: 33392191 PMC7773823

[35] Hanahan D . Hallmarks of cancer: new dimensions. Cancer Discov. (2022) 12:31–46. doi: 10.1158/2159-8290.CD-21-1059, PMID: 35022204

[36] Nance DM Sanders VM . Autonomic innervation and regulation of the immune system (1987-2007). Brain Behav Immun. (2007) 21:736–45. doi: 10.1016/j.bbi.2007.03.008, PMID: 17467231 PMC1986730

[37] Capellino S Claus M Watzl C . Regulation of natural killer cell activity by glucocorticoids, serotonin, dopamine, and epinephrine. Cell Mol Immunol. (2020) 17:705–11. doi: 10.1038/s41423-020-0477-9, PMID: 32503998 PMC7331581

[38] Rosas-Ballina M Olofsson PS Ochani M Valdes-Ferrer SI Levine YA Reardon C . Acetylcholine-synthesizing T cells relay neural signals in a vagus nerve circuit. Science. (2011) 334:98–101. doi: 10.1126/science.1209985, PMID: 21921156 PMC4548937

[39] Wang H Yu M Ochani M Amella CA Tanovic M Susarla S . Nicotinic acetylcholine receptor alpha7 subunit is an essential regulator of inflammation. Nature. (2003) 421:384–8. doi: 10.1038/nature01339, PMID: 12508119

[40] Qin JF Jin FJ Li N Guan HT Lan L Ni H . Adrenergic receptor beta2 activation by stress promotes breast cancer progression through macrophages M2 polarization in tumor microenvironment. BMB Rep. (2015) 48:295–300. doi: 10.5483/bmbrep.2015.48.5.008, PMID: 25748171 PMC4578570

[41] Sloan EK Priceman SJ Cox BF Yu S Pimentel MA Tangkanangnukul V . The sympathetic nervous system induces a metastatic switch in primary breast cancer. Cancer Res. (2010) 70:7042–52. doi: 10.1158/0008-5472.CAN-10-0522, PMID: 20823155 PMC2940980

[42] Mohammadpour H O’Neil R Qiu J McCarthy PL Repasky EA Cao X . Blockade of host beta2-adrenergic receptor enhances graft-versus-tumor effect through modulating apcs. J Immunol. (2018) 200:2479–88. doi: 10.4049/jimmunol.1701752, PMID: 29445008 PMC5860988

[43] Ueshima H Inada T Shingu K . Suppression of phagosome proteolysis and matrigel migration with the alpha2-adrenergic receptor agonist dexmedetomidine in murine dendritic cells. Immunopharmacol Immunotoxicol. (2013) 35:558–66. doi: 10.3109/08923973.2013.822509, PMID: 23927488

[44] Nissen MD Sloan EK Mattarollo SR . Beta-adrenergic signaling impairs antitumor cd8(+) T-cell responses to B-cell lymphoma immunotherapy. Cancer Immunol Res. (2018) 6:98–109. doi: 10.1158/2326-6066.CIR-17-0401, PMID: 29146881

[45] Qiao G Bucsek MJ Winder NM Chen M Giridharan T Olejniczak SH . Beta-adrenergic signaling blocks murine cd8(+) T-cell metabolic reprogramming during activation: A mechanism for immunosuppression by adrenergic stress. Cancer Immunol Immunother. (2019) 68:11–22. doi: 10.1007/s00262-018-2243-8, PMID: 30229289 PMC6326964

[46] Bucsek MJ Qiao G MacDonald CR Giridharan T Evans L Niedzwecki B . Beta-adrenergic signaling in mice housed at standard temperatures suppresses an effector phenotype in cd8(+) T cells and undermines checkpoint inhibitor therapy. Cancer Res. (2017) 77:5639–51. doi: 10.1158/0008-5472.CAN-17-0546, PMID: 28819022 PMC5645237

[47] Mohammadpour H MacDonald CR Qiao G Chen M Dong B Hylander BL . Beta2 adrenergic receptor-mediated signaling regulates the immunosuppressive potential of myeloid-derived suppressor cells. J Clin Invest. (2019) 129:5537–52. doi: 10.1172/JCI129502, PMID: 31566578 PMC6877316

[48] Nakai A Hayano Y Furuta F Noda M Suzuki K . Control of lymphocyte egress from lymph nodes through beta2-adrenergic receptors. J Exp Med. (2014) 211:2583–98. doi: 10.1084/jem.20141132, PMID: 25422496 PMC4267238

[49] Nevin JT Moussa M Corwin WL Mandoiu II Srivastava PK . Sympathetic nervous tone limits the development of myeloid-derived suppressor cells. Sci Immunol. (2020) 5:eaay9368. doi: 10.1126/sciimmunol.aay9368, PMID: 32917793

[50] Dubeykovskaya Z Si Y Chen X Worthley DL Renz BW Urbanska AM . Neural innervation stimulates splenic tff2 to arrest myeloid cell expansion and cancer. Nat Commun. (2016) 7:10517. doi: 10.1038/ncomms10517, PMID: 26841680 PMC4742920

[51] Keskinov AA Tapias V Watkins SC Ma Y Shurin MR Shurin GV . Impact of the sensory neurons on melanoma growth *in vivo*. PloS One. (2016) 11:e0156095. doi: 10.1371/journal.pone.0156095, PMID: 27227315 PMC4882065

[52] Fousek K Horn LA Palena C . Interleukin-8: A chemokine at the intersection of cancer plasticity, angiogenesis, and immune suppression. Pharmacol Ther. (2021) 219:107692. doi: 10.1016/j.pharmthera.2020.107692, PMID: 32980444 PMC8344087

[53] Soler MF Abaurrea A Azcoaga P Araujo AM Caffarel MM . New perspectives in cancer immunotherapy: targeting il-6 cytokine family. J Immunother Cancer. (2023) 11:e007530. doi: 10.1136/jitc-2023-007530, PMID: 37945321 PMC10649711

[54] Cole SW Sood AK . Molecular pathways: beta-adrenergic signaling in cancer. Clin Cancer Res. (2012) 18:1201–6. doi: 10.1158/1078-0432.CCR-11-0641, PMID: 22186256 PMC3294063

[55] Basbaum AI Bautista DM Scherrer G Julius D . Cellular and molecular mechanisms of pain. Cell. (2009) 139:267–84. doi: 10.1016/j.cell.2009.09.028, PMID: 19837031 PMC2852643

[56] Peters CM Ghilardi JR Keyser CP Kubota K Lindsay TH Luger NM . Tumor-induced injury of primary afferent sensory nerve fibers in bone cancer pain. Exp Neurol. (2005) 193:85–100. doi: 10.1016/j.expneurol.2004.11.028, PMID: 15817267

[57] Ghilardi JR Rohrich H Lindsay TH Sevcik MA Schwei MJ Kubota K . Selective blockade of the capsaicin receptor trpv1 attenuates bone cancer pain. J Neurosci. (2005) 25:3126–31. doi: 10.1523/JNEUROSCI.3815-04.2005, PMID: 15788769 PMC6725088

[58] Mantyh PW Clohisy DR Koltzenburg M Hunt SP . Molecular mechanisms of cancer pain. Nat Rev Cancer. (2002) 2:201–9. doi: 10.1038/nrc747, PMID: 11990856

[59] Cohnen J Kornstadt L Hahnefeld L Ferreiros N Pierre S Koehl U . Tumors provoke inflammation and perineural microlesions at adjacent peripheral nerves. Cells. (2020) 9:320. doi: 10.3390/cells9020320, PMID: 32013137 PMC7072456

[60] Mantyh PW . Cancer pain and its impact on diagnosis, survival and quality of life. Nat Rev Neurosci. (2006) 7:797–809. doi: 10.1038/nrn1914, PMID: 16988655

[61] Medzhitov R . Origin and physiological roles of inflammation. Nature. (2008) 454:428–35. doi: 10.1038/nature07201, PMID: 18650913

[62] Liu J Chen Y Chen G . The role and mechanisms of macrophages in chronic pain: A peripheral-to-central perspective. Brain Res Bull. (2025) 229:111470. doi: 10.1016/j.brainresbull.2025.111470, PMID: 40683471

[63] Vendrell I Macedo D Alho I Dionisio MR Costa L . Treatment of cancer pain by targeting cytokines. Mediators Inflammation. (2015) 2015:984570. doi: 10.1155/2015/984570, PMID: 26538839 PMC4619962

[64] Ji RR Nackley A Huh Y Terrando N Maixner W . Neuroinflammation and central sensitization in chronic and widespread pain. Anesthesiology. (2018) 129:343–66. doi: 10.1097/ALN.0000000000002130, PMID: 29462012 PMC6051899

[65] Santoni A Mercadante S Arcuri E . Chronic cancer and non-cancer pain and opioid-induced hyperalgesia share common mechanisms: neuroinflammation and central sensitization. Minerva Anestesiol. (2021) 87:210–22. doi: 10.23736/S0375-9393.20.14822-3, PMID: 33300326

[66] Stein C Clark JD Oh U Vasko MR Wilcox GL Overland AC . Peripheral mechanisms of pain and analgesia. Brain Res Rev. (2009) 60:90–113. doi: 10.1016/j.brainresrev.2008.12.017, PMID: 19150465 PMC2730351

[67] Stein C Schafer M Machelska H . Attacking pain at its source: new perspectives on opioids. Nat Med. (2003) 9:1003–8. doi: 10.1038/nm908, PMID: 12894165

[68] Hutchinson MR Coats BD Lewis SS Zhang Y Sprunger DB Rezvani N . Proinflammatory cytokines oppose opioid-induced acute and chronic analgesia. Brain Behav Immun. (2008) 22:1178–89. doi: 10.1016/j.bbi.2008.05.004, PMID: 18599265 PMC2783238

[69] Hutchinson MR Zhang Y Shridhar M Evans JH Buchanan MM Zhao TX . Evidence that opioids may have toll-like receptor 4 and md-2 effects. Brain Behav Immun. (2010) 24:83–95. doi: 10.1016/j.bbi.2009.08.004, PMID: 19679181 PMC2788078

[70] Wang X Loram LC Ramos K de Jesus AJ Thomas J Cheng K . Morphine activates neuroinflammation in a manner parallel to endotoxin. Proc Natl Acad Sci U.S.A. (2012) 109:6325–30. doi: 10.1073/pnas.1200130109, PMID: 22474354 PMC3341002

[71] Arcuri E Mercadante S Santoni A . Immunity and pain: is it time for the birth of immunoalgology? Minerva Anestesiol. (2021) 87:845–7. doi: 10.23736/S0375-9393.21.15713-X, PMID: 34036770

[72] Santoni A Arcuri E . The ambiguity of opioids revealed by immunology is changing the knowledge and the therapeutic approach in cancer and non-cancer pain: A narrative review. Immunol Lett. (2020) 226:12–21. doi: 10.1016/j.imlet.2020.06.011, PMID: 32590120

[73] Santoni A Santoni M Arcuri E . Chronic cancer pain: opioids within tumor microenvironment affect neuroinflammation, tumor and pain evolution. Cancers (Basel). (2022) 14:2253. doi: 10.3390/cancers14092253, PMID: 35565382 PMC9104169

[74] Schmidt BL Hamamoto DT Simone DA Wilcox GL . Mechanism of cancer pain. Mol Interv. (2010) 10:164–78. doi: 10.1124/mi.10.3.7, PMID: 20539035 PMC2895277

[75] Doyle TM Salvemini D . Mini-review: mitochondrial dysfunction and chemotherapy-induced neuropathic pain. Neurosci Lett. (2021) 760:136087. doi: 10.1016/j.neulet.2021.136087, PMID: 34182057 PMC9260825

[76] Calcinotto A Kohli J Zagato E Pellegrini L Demaria M Alimonti A . Cellular senescence: aging, cancer, and injury. Physiol Rev. (2019) 99:1047–78. doi: 10.1152/physrev.00020.2018, PMID: 30648461

[77] Gorgoulis V Adams PD Alimonti A Bennett DC Bischof O Bishop C . Cellular senescence: defining a path forward. Cell. (2019) 179:813–27. doi: 10.1016/j.cell.2019.10.005, PMID: 31675495

[78] Eggert T Wolter K Ji J Ma C Yevsa T Klotz S . Distinct functions of senescence-associated immune responses in liver tumor surveillance and tumor progression. Cancer Cell. (2016) 30:533–47. doi: 10.1016/j.ccell.2016.09.003, PMID: 27728804 PMC7789819

[79] Schmitt CA Wang B Demaria M . Senescence and cancer - role and therapeutic opportunities. Nat Rev Clin Oncol. (2022) 19:619–36. doi: 10.1038/s41571-022-00668-4, PMID: 36045302 PMC9428886

[80] Guan X LaPak KM Hennessey RC Yu CY Shakya R Zhang J . Stromal senescence by prolonged cdk4/6 inhibition potentiates tumor growth. Mol Cancer Res. (2017) 15:237–49. doi: 10.1158/1541-7786.MCR-16-0319, PMID: 28039358 PMC5334447

[81] Kanehira M Fujiwara T Nakajima S Okitsu Y Onishi Y Fukuhara N . An lysophosphatidic acid receptors 1 and 3 axis governs cellular senescence of mesenchymal stromal cells and promotes growth and vascularization of multiple myeloma. Stem Cells. (2017) 35:739–53. doi: 10.1002/stem.2499, PMID: 27641212

[82] Pardella E Pranzini E Nesi I Parri M Spatafora P Torre E . Therapy-induced stromal senescence promoting aggressiveness of prostate and ovarian cancer. Cells. (2022) 11:(24):4026. doi: 10.3390/cells11244026, PMID: 36552790 PMC9776582

[83] Ruhland MK Loza AJ Capietto AH Luo X Knolhoff BL Flanagan KC . Stromal senescence establishes an immunosuppressive microenvironment that drives tumorigenesis. Nat Commun. (2016) 7:11762. doi: 10.1038/ncomms11762, PMID: 27272654 PMC4899869

[84] Antonangeli F Zingoni A Soriani A Santoni A . Senescent cells: living or dying is a matter of nk cells. J Leukoc Biol. (2019) 105:1275–83. doi: 10.1002/JLB.MR0718-299R, PMID: 30811627

[85] Prata L Ovsyannikova IG Tchkonia T Kirkland JL . Senescent cell clearance by the immune system: emerging therapeutic opportunities. Semin Immunol. (2018) 40:101275. doi: 10.1016/j.smim.2019.04.003, PMID: 31088710 PMC7061456

[86] Chibaya L Snyder J Ruscetti M . Senescence and the tumor-immune landscape: implications for cancer immunotherapy. Semin Cancer Biol. (2022) 86:827–45. doi: 10.1016/j.semcancer.2022.02.005, PMID: 35143990 PMC9357237

[87] Antelo-Iglesias L Picallos-Rabina P Estevez-Souto V Da Silva-Alvarez S Collado M . The role of cellular senescence in tissue repair and regeneration. Mech Ageing Dev. (2021) 198:111528. doi: 10.1016/j.mad.2021.111528, PMID: 34181964

[88] Wang B Han J Elisseeff JH Demaria M . The senescence-associated secretory phenotype and its physiological and pathological implications. Nat Rev Mol Cell Biol. (2024) 25:958–78. doi: 10.1038/s41580-024-00727-x, PMID: 38654098

[89] Yun MH . Cellular senescence in tissue repair: every cloud has a silver lining. Int J Dev Biol. (2018) 62:591–604. doi: 10.1387/ijdb.180081my, PMID: 29938770

[90] Dunn GP Bruce AT Ikeda H Old LJ Schreiber RD . Cancer immunoediting: from immunosurveillance to tumor escape. Nat Immunol. (2002) 3:991–8. doi: 10.1038/ni1102-991, PMID: 12407406

[91] Zingoni A Antonangeli F Sozzani S Santoni A Cippitelli M Soriani A . The senescence journey in cancer immunoediting. Mol Cancer. (2024) 23:68. doi: 10.1186/s12943-024-01973-5, PMID: 38561826 PMC10983694

[92] Hernandez-Segura A de Jong TV Melov S Guryev V Campisi J Demaria M . Unmasking transcriptional heterogeneity in senescent cells. Curr Biol. (2017) 27:2652–60 e4. doi: 10.1016/j.cub.2017.07.033, PMID: 28844647 PMC5788810

[93] Ito Y Hoare M Narita M . Spatial and temporal control of senescence. Trends Cell Biol. (2017) 27:820–32. doi: 10.1016/j.tcb.2017.07.004, PMID: 28822679

[94] Kumari R Jat P . Mechanisms of cellular senescence: cell cycle arrest and senescence associated secretory phenotype. Front Cell Dev Biol. (2021) 9:645593. doi: 10.3389/fcell.2021.645593, PMID: 33855023 PMC8039141

[95] Schweizerhof M Stosser S Kurejova M Njoo C Gangadharan V Agarwal N . Hematopoietic colony-stimulating factors mediate tumor-nerve interactions and bone cancer pain. Nat Med. (2009) 15:802–7. doi: 10.1038/nm.1976, PMID: 19525966

[96] Coppe JP Desprez PY Krtolica A Campisi J . The senescence-associated secretory phenotype: the dark side of tumor suppression. Annu Rev Pathol. (2010) 5:99–118. doi: 10.1146/annurev-pathol-121808-102144, PMID: 20078217 PMC4166495

[97] Coppe JP Patil CK Rodier F Sun Y Munoz DP Goldstein J . Senescence-associated secretory phenotypes reveal cell-nonautonomous functions of oncogenic ras and the P53 tumor suppressor. PloS Biol. (2008) 6:2853–68. doi: 10.1371/journal.pbio.0060301, PMID: 19053174 PMC2592359

[98] Fang D Kong LY Cai J Li S Liu XD Han JS . Interleukin-6-mediated functional upregulation of trpv1 receptors in dorsal root ganglion neurons through the activation of jak/pi3k signaling pathway: roles in the development of bone cancer pain in a rat model. Pain. (2015) 156:1124–44. doi: 10.1097/j.pain.0000000000000158, PMID: 25775359

[99] Malek N Pajak A Kolosowska N Kucharczyk M Starowicz K . The importance of trpv1-sensitisation factors for the development of neuropathic pain. Mol Cell Neurosci. (2015) 65:1–10. doi: 10.1016/j.mcn.2015.02.001, PMID: 25662734

[100] Zhou YQ Liu Z Liu ZH Chen SP Li M Shahveranov A . Interleukin-6: an emerging regulator of pathological pain. J Neuroinflamm. (2016) 13:141. doi: 10.1186/s12974-016-0607-6, PMID: 27267059 PMC4897919

[101] Ramer MS Murphy PG Richardson PM Bisby MA . Spinal nerve lesion-induced mechanoallodynia and adrenergic sprouting in sensory ganglia are attenuated in interleukin-6 knockout mice. Pain. (1998) 78:115–21. doi: 10.1016/S0304-3959(98)00121-3, PMID: 9839821

[102] Melemedjian OK Asiedu MN Tillu DV Peebles KA Yan J Ertz N . Il-6- and ngf-induced rapid control of protein synthesis and nociceptive plasticity via convergent signaling to the eif4f complex. J Neurosci. (2010) 30:15113–23. doi: 10.1523/JNEUROSCI.3947-10.2010, PMID: 21068317 PMC3056511

[103] Melemedjian OK Tillu DV Moy JK Asiedu MN Mandell EK Ghosh S . Local translation and retrograde axonal transport of creb regulates il-6-induced nociceptive plasticity. Mol Pain. (2014) 10:45. doi: 10.1186/1744-8069-10-45, PMID: 24993495 PMC4091745

[104] Uceyler N Kafke W Riediger N He L Necula G Toyka KV . Elevated proinflammatory cytokine expression in affected skin in small fiber neuropathy. Neurology. (2010) 74:1806–13. doi: 10.1212/WNL.0b013e3181e0f7b3, PMID: 20513817

[105] Callizot N Andriambeloson E Glass J Revel M Ferro P Cirillo R . Interleukin-6 Protects against Paclitaxel, Cisplatin and Vincristine-Induced Neuropathies without Impairing Chemotherapeutic Activity. Cancer Chemother Pharmacol. (2008) 62:995–1007. doi: 10.1007/s00280-008-0689-7, PMID: 18270703

[106] Kiguchi N Maeda T Kobayashi Y Kondo T Ozaki M Kishioka S . The critical role of invading peripheral macrophage-derived interleukin-6 in vincristine-induced mechanical allodynia in mice. Eur J Pharmacol. (2008) 592:87–92. doi: 10.1016/j.ejphar.2008.07.008, PMID: 18652822

[107] Al-Mazidi S Farhat K Nedjadi T Chaudhary A Zin Al-Abdin O Rabah D . Association of interleukin-6 and other cytokines with self-reported pain in prostate cancer patients receiving chemotherapy. Pain Med. (2018) 19:1058–66. doi: 10.1093/pm/pnx145, PMID: 29016954

[108] Starkweather A . Increased interleukin-6 activity associated with painful chemotherapy-induced peripheral neuropathy in women after breast cancer treatment. Nurs Res Pract. (2010) 2010:281531. doi: 10.1155/2010/281531, PMID: 21994811 PMC3168945

[109] Childs BG Gluscevic M Baker DJ Laberge RM Marquess D Dananberg J . Senescent cells: an emerging target for diseases of ageing. Nat Rev Drug Discov. (2017) 16:718–35. doi: 10.1038/nrd.2017.116, PMID: 28729727 PMC5942225

[110] van Deursen JM . The role of senescent cells in ageing. Nature. (2014) 509:439–46. doi: 10.1038/nature13193, PMID: 24848057 PMC4214092

[111] Iannello A Thompson TW Ardolino M Lowe SW Raulet DH . P53-dependent chemokine production by senescent tumor cells supports nkg2d-dependent tumor elimination by natural killer cells. J Exp Med. (2013) 210:2057–69. doi: 10.1084/jem.20130783, PMID: 24043758 PMC3782044

[112] Abbadie C LIndia JA Cumiskey AM Peterson LB Mudgett JS Bayne EK . Impaired neuropathic pain responses in mice lacking the chemokine receptor ccr2. Proc Natl Acad Sci U.S.A. (2003) 100:7947–52. doi: 10.1073/pnas.1331358100, PMID: 12808141 PMC164693

[113] Oh SB Tran PB Gillard SE Hurley RW Hammond DL Miller RJ . Chemokines and glycoprotein120 produce pain hypersensitivity by directly exciting primary nociceptive neurons. J Neurosci. (2001) 21:5027–35. doi: 10.1523/JNEUROSCI.21-14-05027.2001, PMID: 11438578 PMC6762869

[114] Tanaka T Minami M Nakagawa T Satoh M . Enhanced production of monocyte chemoattractant protein-1 in the dorsal root ganglia in a rat model of neuropathic pain: possible involvement in the development of neuropathic pain. Neurosci Res. (2004) 48:463–9. doi: 10.1016/j.neures.2004.01.004, PMID: 15041200

[115] Acosta JC Banito A Wuestefeld T Georgilis A Janich P Morton JP . A complex secretory program orchestrated by the inflammasome controls paracrine senescence. Nat Cell Biol. (2013) 15:978–90. doi: 10.1038/ncb2784, PMID: 23770676 PMC3732483

[116] Di Mitri D Alimonti A . Non-cell-autonomous regulation of cellular senescence in cancer. Trends Cell Biol. (2016) 26:215–26. doi: 10.1016/j.tcb.2015.10.005, PMID: 26564316

[117] Orjalo AV Bhaumik D Gengler BK Scott GK Campisi J . Cell surface-bound il-1alpha is an upstream regulator of the senescence-associated il-6/il-8 cytokine network. Proc Natl Acad Sci U.S.A. (2009) 106:17031–6. doi: 10.1073/pnas.0905299106, PMID: 19805069 PMC2761322

[118] Zhang J Wang L Wang H Su Z Pang X . Neuroinflammation and central pi3k/akt/mtor signal pathway contribute to bone cancer pain. Mol Pain. (2019) 15:1744806919830240. doi: 10.1177/1744806919830240, PMID: 30717619 PMC6390230

[119] Zhang RX Liu B Li A Wang L Ren K Qiao JT . Interleukin 1beta facilitates bone cancer pain in rats by enhancing nmda receptor nr-1 subunit phosphorylation. Neuroscience. (2008) 154:1533–8. doi: 10.1016/j.neuroscience.2008.04.072, PMID: 18554806 PMC2495055

[120] Wiley CD Brumwell AN Davis SS Jackson JR Valdovinos A Calhoun C . Secretion of leukotrienes by senescent lung fibroblasts promotes pulmonary fibrosis. JCI Insight. (2019) 4:e130056. doi: 10.1172/jci.insight.130056, PMID: 31687975 PMC6975274

[121] Zdanov S Bernard D Debacq-Chainiaux F Martien S Gosselin K Vercamer C . Normal or stress-induced fibroblast senescence involves cox-2 activity. Exp Cell Res. (2007) 313:3046–56. doi: 10.1016/j.yexcr.2007.04.033, PMID: 17560572

[122] Narzt MS Pils V Kremslehner C Nagelreiter IM Schosserer M Bessonova E . Epilipidomics of senescent dermal fibroblasts identify lysophosphatidylcholines as pleiotropic senescence-associated secretory phenotype (Sasp) factors. J Invest Dermatol. (2021) 141:993–1006 e15. doi: 10.1016/j.jid.2020.11.020, PMID: 33333126

[123] Pils V Terlecki-Zaniewicz L Schosserer M Grillari J Lammermann I . The role of lipid-based signalling in wound healing and senescence. Mech Ageing Dev. (2021) 198:111527. doi: 10.1016/j.mad.2021.111527, PMID: 34174292

[124] Chinta SJ Lieu CA Demaria M Laberge RM Campisi J Andersen JK . Environmental stress, ageing and glial cell senescence: A novel mechanistic link to parkinson’s disease? J Intern Med. (2013) 273:429–36. doi: 10.1111/joim.12029, PMID: 23600398 PMC3633085

[125] Sapieha P Mallette FA . Cellular senescence in postmitotic cells: beyond growth arrest. Trends Cell Biol. (2018) 28:595–607. doi: 10.1016/j.tcb.2018.03.003, PMID: 29704982

[126] Ma Y Farny NG . Connecting the dots: neuronal senescence, stress granules, and neurodegeneration. Gene. (2023) 871:147437. doi: 10.1016/j.gene.2023.147437, PMID: 37084987 PMC10205695

[127] Sah E Krishnamurthy S Ahmidouch MY Gillispie GJ Milligan C Orr ME . The cellular senescence stress response in post-mitotic brain cells: cell survival at the expense of tissue degeneration. Life (Basel). (2021) 11:229. doi: 10.3390/life11030229, PMID: 33799628 PMC7998276

[128] Musi N Valentine JM Sickora KR Baeuerle E Thompson CS Shen Q . Tau protein aggregation is associated with cellular senescence in the brain. Aging Cell. (2018) 17:e12840. doi: 10.1111/acel.12840, PMID: 30126037 PMC6260915

[129] Moreno-Blas D Gorostieta-Salas E Pommer-Alba A Mucino-Hernandez G Geronimo-Olvera C Maciel-Baron LA . Cortical neurons develop a senescence-like phenotype promoted by dysfunctional autophagy. Aging (Albany NY). (2019) 11:6175–98. doi: 10.18632/aging.102181, PMID: 31469660 PMC6738425

[130] Ishikawa S Ishikawa F . Proteostasis failure and cellular senescence in long-term cultured postmitotic rat neurons. Aging Cell. (2020) 19:e13071. doi: 10.1111/acel.13071, PMID: 31762159 PMC6974705

[131] Jurk D Wang C Miwa S Maddick M Korolchuk V Tsolou A . Postmitotic neurons develop a P21-dependent senescence-like phenotype driven by a DNA damage response. Aging Cell. (2012) 11:996–1004. doi: 10.1111/j.1474-9726.2012.00870.x, PMID: 22882466 PMC3533793

[132] Calls A Torres-Espin A Navarro X Yuste VJ Udina E Bruna J . Cisplatin-induced peripheral neuropathy is associated with neuronal senescence-like response. Neuro Oncol. (2021) 23:88–99. doi: 10.1093/neuonc/noaa151, PMID: 32597980 PMC7850121

[133] Acklin S Zhang M Du W Zhao X Plotkin M Chang J . Depletion of senescent-like neuronal cells alleviates cisplatin-induced peripheral neuropathy in mice. Sci Rep. (2020) 10:14170. doi: 10.1038/s41598-020-71042-6, PMID: 32843706 PMC7447787

[134] Chang J Wang Y Shao L Laberge RM Demaria M Campisi J . Clearance of senescent cells by abt263 rejuvenates aged hematopoietic stem cells in mice. Nat Med. (2016) 22:78–83. doi: 10.1038/nm.4010, PMID: 26657143 PMC4762215

[135] Borghesan M Hoogaars WMH Varela-Eirin M Talma N Demaria M . A senescence-centric view of aging: implications for longevity and disease. Trends Cell Biol. (2020) 30:777–91. doi: 10.1016/j.tcb.2020.07.002, PMID: 32800659

[136] Chaib S Tchkonia T Kirkland JL . Cellular senescence and senolytics: the path to the clinic. Nat Med. (2022) 28:1556–68. doi: 10.1038/s41591-022-01923-y, PMID: 35953721 PMC9599677

[137] Muralidharan A Sotocinal SG Yousefpour N Akkurt N Lima LV Tansley S . Long-term male-specific chronic pain via telomere- and P53−Mediated spinal cord cellular senescence. J Clin Invest. (2022) 132:e151817. doi: 10.1172/JCI151817, PMID: 35426375 PMC9012275

[138] Zhang WJ Wu CL Liu JP . Schwann cells as a target cell for the treatment of cancer pain. Glia. (2023) 71:2309–22. doi: 10.1002/glia.24391, PMID: 37218574

[139] Wei Z Fei Y Su W Chen G . Emerging role of schwann cells in neuropathic pain: receptors, glial mediators and myelination. Front Cell Neurosci. (2019) 13:116. doi: 10.3389/fncel.2019.00116, PMID: 30971897 PMC6445947

[140] Marinelli S Nazio F Tinari A Ciarlo L D’Amelio M Pieroni L . Schwann cell autophagy counteracts the onset and chronification of neuropathic pain. Pain. (2014) 155:93–107. doi: 10.1016/j.pain.2013.09.013, PMID: 24041962

[141] Sommer C Schafers M . Painful mononeuropathy in C57bl/wld mice with delayed wallerian degeneration: differential effects of cytokine production and nerve regeneration on thermal and mechanical hypersensitivity. Brain Res. (1998) 784:154–62. doi: 10.1016/s0006-8993(97)01327-9, PMID: 9518588

[142] De Logu F Marini M Landini L Souza Monteiro de Araujo D Bartalucci N Trevisan G . Peripheral nerve resident macrophages and schwann cells mediate cancer-induced pain. Cancer Res. (2021) 81:3387–401. doi: 10.1158/0008-5472.CAN-20-3326, PMID: 33771895 PMC8260461

[143] De Logu F Nassini R Materazzi S Carvalho Goncalves M Nosi D Rossi Degl’Innocenti D . Schwann cell trpa1 mediates neuroinflammation that sustains macrophage-dependent neuropathic pain in mice. Nat Commun. (2017) 8:1887. doi: 10.1038/s41467-017-01739-2, PMID: 29192190 PMC5709495

[144] Salvo E Saraithong P Curtin JG Janal MN Ye Y . Reciprocal interactions between cancer and schwann cells contribute to oral cancer progression and pain. Heliyon. (2019) 5:e01223. doi: 10.1016/j.heliyon.2019.e01223, PMID: 30815600 PMC6378335

[145] Salvo E Tu NH Scheff NN Dubeykovskaya ZA Chavan SA Aouizerat BE . Tnfalpha promotes oral cancer growth, pain, and schwann cell activation. Sci Rep. (2021) 11:1840. doi: 10.1038/s41598-021-81500-4, PMID: 33469141 PMC7815837

[146] Fuentes-Flores A Geronimo-Olvera C Girardi K Necunir-Ibarra D Patel SK Bons J . Senescent schwann cells induced by aging and chronic denervation impair axonal regeneration following peripheral nerve injury. EMBO Mol Med. (2023) 15:e17907. doi: 10.15252/emmm.202317907, PMID: 37860842 PMC10701627

[147] Kohlmeyer JL Kaemmer CA Umesalma S Gourronc FA Klingelhutz AJ Quelle DE . Rabl6a regulates schwann cell senescence in an rb1-dependent manner. Int J Mol Sci. (2021) 22:5367. doi: 10.3390/ijms22105367, PMID: 34065204 PMC8161079

[148] Saheb-Al-Zamani M Yan Y Farber SJ Hunter DA Newton P Wood MD . Limited regeneration in long acellular nerve allografts is associated with increased schwann cell senescence. Exp Neurol. (2013) 247:165–77. doi: 10.1016/j.expneurol.2013.04.011, PMID: 23644284 PMC3863361

[149] Jain A Hakim S Woolf CJ . Immune drivers of physiological and pathological pain. J Exp Med. (2024) 221:e20221687. doi: 10.1084/jem.20221687, PMID: 38607420 PMC11010323

[150] Ren K Dubner R . Interactions between the immune and nervous systems in pain. Nat Med. (2010) 16:1267–76. doi: 10.1038/nm.2234, PMID: 20948535 PMC3077564

[151] Hakim S Jain A Woolf CJ . Immune drivers of pain resolution and protection. Nat Immunol. (2024) 25:2200–8. doi: 10.1038/s41590-024-02002-9, PMID: 39528810

[152] Laumet G Bavencoffe A Edralin JD Huo XJ Walters ET Dantzer R . Interleukin-10 resolves pain hypersensitivity induced by cisplatin by reversing sensory neuron hyperexcitability. Pain. (2020) 161:2344–52. doi: 10.1097/j.pain.0000000000001921, PMID: 32427749 PMC7962468

[153] Celik MO Labuz D Keye J Glauben R Machelska H . Il-4 induces M2 macrophages to produce sustained analgesia via opioids. JCI Insight. (2020) 5:e133093. doi: 10.1172/jci.insight.133093, PMID: 32102987 PMC7101153

[154] Noguchi M Yoshita M Sakai K Matsumoto Y Arahata M Ontachi Y . Peripheral neuropathy associated with chronic natural killer cell lymphocytosis. J Neurol Sci. (2005) 232:119–22. doi: 10.1016/j.jns.2005.01.013, PMID: 15850593

[155] Davies AJ Rinaldi S Costigan M Oh SB . Cytotoxic immunity in peripheral nerve injury and pain. Front Neurosci. (2020) 14:142. doi: 10.3389/fnins.2020.00142, PMID: 32153361 PMC7047751

[156] Parisien M Lima LV Dagostino C El-Hachem N Drury GL Grant AV . Acute inflammatory response via neutrophil activation protects against the development of chronic pain. Sci Transl Med. (2022) 14:eabj9954. doi: 10.1126/scitranslmed.abj9954, PMID: 35544595 PMC10317000

[157] Greisen J Hokland M Grofte T Hansen PO Jensen TS Vilstrup H . Acute pain induces an instant increase in natural killer cell cytotoxicity in humans and this response is abolished by local anaesthesia. Br J Anaesth. (1999) 83:235–40. doi: 10.1093/bja/83.2.235, PMID: 10618935

[158] Sharify A Mahmoudi M Izad MH Hosseini MJ Sharify M . Effect of acute pain on splenic nk cell activity, lymphocyte proliferation and cytokine production activities. Immunopharmacol Immunotoxicol. (2007) 29:465–76. doi: 10.1080/08923970701619877, PMID: 18075858

[159] Lassen J Sturner KH Gierthmuhlen J Dargvainiene J Kixmuller D Leypoldt F . Protective role of natural killer cells in neuropathic pain conditions. Pain. (2021) 162:2366–75. doi: 10.1097/j.pain.0000000000002274, PMID: 33769361

[160] Gao YH Wang JY Qiao LN Chen SP Tan LH Xu QL . Nk cells mediate the cumulative analgesic effect of electroacupuncture in a rat model of neuropathic pain. BMC Complement Altern Med. (2014) 14:316. doi: 10.1186/1472-6882-14-316, PMID: 25158599 PMC4152576

[161] Kim CK Choi GS Oh SD Han JB Kim SK Ahn HJ . Electroacupuncture up-Regulates Natural Killer Cell Activity Identification of Genes Altering Their Expressions in Electroacupuncture Induced up-Regulation of Natural Killer Cell Activity. J Neuroimmunol. (2005) 168:144–53. doi: 10.1016/j.jneuroim.2005.07.005, PMID: 16154208

[162] Llobet Rosell A Neukomm LJ . Axon death signalling in wallerian degeneration among species and in disease. Open Biol. (2019) 9:190118. doi: 10.1098/rsob.190118, PMID: 31455157 PMC6731592

[163] Hickey WF Ueno K Hiserodt JC Schmidt RE . Exogenously-induced, natural killer cell-mediated neuronal killing: A novel pathogenetic mechanism. J Exp Med. (1992) 176:811–7. doi: 10.1084/jem.176.3.811, PMID: 1512544 PMC2119372

[164] Thygesen P Hougen HP Christensen HB Rygaard J Svendsen O Juul P . Identification of the mononuclear cell infiltrate in the superior cervical ganglion of athymic nude and euthymic rats after guanethidine-induced sympathectomy. Int J Immunopharmacol. (1990) 12:327–30. doi: 10.1016/0192-0561(90)90089-6, PMID: 2184138

[165] Backstrom E Chambers BJ Kristensson K Ljunggren HG . Direct nk cell-mediated lysis of syngenic dorsal root ganglia neurons *in vitro*. J Immunol. (2000) 165:4895–900. doi: 10.4049/jimmunol.165.9.4895, PMID: 11046014

[166] Davies AJ Kim HW Gonzalez-Cano R Choi J Back SK Roh SE . Natural killer cells degenerate intact sensory afferents following nerve injury. Cell. (2019) 176:716–28 e18. doi: 10.1016/j.cell.2018.12.022, PMID: 30712871 PMC6418410

[167] Costigan M Scholz J Woolf CJ . Neuropathic pain: A maladaptive response of the nervous system to damage. Annu Rev Neurosci. (2009) 32:1–32. doi: 10.1146/annurev.neuro.051508.135531, PMID: 19400724 PMC2768555

[168] Xie W Strong JA Zhang JM . Active nerve regeneration with failed target reinnervation drives persistent neuropathic pain. eNeuro. (2017) 4. doi: 10.1523/ENEURO.0008-17.2017, PMID: 28197545 PMC5290455

[169] Kim HW Wang S Davies AJ Oh SB . The therapeutic potential of natural killer cells in neuropathic pain. Trends Neurosci. (2023) 46:617–27. doi: 10.1016/j.tins.2023.05.008, PMID: 37385878

[170] Brown DC . Resiniferatoxin: the evolution of the “Molecular scalpel” for chronic pain relief. Pharm (Basel). (2016) 9:47. doi: 10.3390/ph9030047, PMID: 27529257 PMC5039500

[171] Gangadharan V Zheng H Taberner FJ Landry J Nees TA Pistolic J . Neuropathic pain caused by miswiring and abnormal end organ targeting. Nature. (2022) 606:137–45. doi: 10.1038/s41586-022-04777-z, PMID: 35614217 PMC9159955

[172] Vulchanova L Olson TH Stone LS Riedl MS Elde R Honda CN . Cytotoxic targeting of isolectin ib4-binding sensory neurons. Neuroscience. (2001) 108:143–55. doi: 10.1016/s0306-4522(01)00377-3, PMID: 11738138

[173] Antonangeli F Soriani A Ricci B Ponzetta A Benigni G Morrone S . Natural killer cell recognition of *in vivo* drug-induced senescent multiple myeloma cells. Oncoimmunology. (2016) 5:e1218105. doi: 10.1080/2162402X.2016.1218105, PMID: 27853638 PMC5087311

[174] Borrelli C Ricci B Vulpis E Fionda C Ricciardi MR Petrucci MT . Drug-induced senescent multiple myeloma cells elicit nk cell proliferation by direct or exosome-mediated il15 trans-presentation. Cancer Immunol Res. (2018) 6:860–9. doi: 10.1158/2326-6066.CIR-17-0604, PMID: 29691234

[175] Ruscetti M Leibold J Bott MJ Fennell M Kulick A Salgado NR . Nk cell-mediated cytotoxicity contributes to tumor control by a cytostatic drug combination. Science. (2018) 362:1416–22. doi: 10.1126/science.aas9090, PMID: 30573629 PMC6711172

[176] Soriani A Zingoni A Cerboni C Iannitto ML Ricciardi MR Di Gialleonardo V . Atm-atr-dependent up-regulation of dnam-1 and nkg2d ligands on multiple myeloma cells by therapeutic agents results in enhanced nk-cell susceptibility and is associated with a senescent phenotype. Blood. (2009) 113:3503–11. doi: 10.1182/blood-2008-08-173914, PMID: 19098271

[177] Xue W Zender L Miething C Dickins RA Hernando E Krizhanovsky V . Senescence and tumour clearance is triggered by P53 restoration in murine liver carcinomas. Nature. (2007) 445:656–60. doi: 10.1038/nature05529, PMID: 17251933 PMC4601097

[178] Sagiv A Biran A Yon M Simon J Lowe SW Krizhanovsky V . Granule exocytosis mediates immune surveillance of senescent cells. Oncogene. (2013) 32:1971–7. doi: 10.1038/onc.2012.206, PMID: 22751116 PMC3630483

[179] Sagiv A Burton DG Moshayev Z Vadai E Wensveen F Ben-Dor S . Nkg2d ligands mediate immunosurveillance of senescent cells. Aging (Albany NY). (2016) 8:328–44. doi: 10.18632/aging.100897, PMID: 26878797 PMC4789586

[180] Backstrom E Chambers BJ Ho EL Naidenko OV Mariotti R Fremont DH . Natural killer cell-mediated lysis of dorsal root ganglia neurons via rae1/nkg2d interactions. Eur J Immunol. (2003) 33:92–100. doi: 10.1002/immu.200390012, PMID: 12594837

[181] Shen YY Zhang RR Liu QY Li SY Yi S . Robust temporal changes of cellular senescence and proliferation after sciatic nerve injury. Neural Regener Res. (2022) 17:1588–95. doi: 10.4103/1673-5374.330619, PMID: 34916445 PMC8771116

[182] Cerboni C Zingoni A Cippitelli M Piccoli M Frati L Santoni A . Antigen-activated human T lymphocytes express cell-surface nkg2d ligands via an atm/atr-dependent mechanism and become susceptible to autologous nk- cell lysis. Blood. (2007) 110:606–15. doi: 10.1182/blood-2006-10-052720, PMID: 17405908

[183] Kawamoto S Hara E . Crosstalk between gut microbiota and cellular senescence: A vicious cycle leading to aging gut. Trends Cell Biol. (2024) 34:626–35. doi: 10.1016/j.tcb.2023.12.004, PMID: 38220548

[184] Lopez-Otin C Blasco MA Partridge L Serrano M Kroemer G . Hallmarks of aging: an expanding universe. Cell. (2023) 186:243–78. doi: 10.1016/j.cell.2022.11.001, PMID: 36599349

[185] Lopez-Otin C Pietrocola F Roiz-Valle D Galluzzi L Kroemer G . Meta-hallmarks of aging and cancer. Cell Metab. (2023) 35:12–35. doi: 10.1016/j.cmet.2022.11.001, PMID: 36599298

[186] D’Alessandro G Antonangeli F Marrocco F Porzia A Lauro C Santoni A . Gut microbiota alterations affect glioma growth and innate immune cells involved in tumor immunosurveillance in mice. Eur J Immunol. (2020) 50:705–11. doi: 10.1002/eji.201948354, PMID: 32034922 PMC7216943

[187] Liang Y Du M Li X Gao J Li Q Li H . Upregulation of lactobacillus spp. In gut microbiota as a novel mechanism for environmental eustress-induced anti-pancreatic cancer effects. Gut Microbes. (2025) 17:2470372. doi: 10.1080/19490976.2025.2470372, PMID: 39988618 PMC11853549

[188] Rizvi ZA Dalal R Sadhu S Kumar Y Kumar S Gupta SK . High-salt diet mediates interplay between nk cells and gut microbiota to induce potent tumor immunity. Sci Adv. (2021) 7:eabg5016. doi: 10.1126/sciadv.abg5016, PMID: 34516769 PMC8442882

[189] Wei H Suo C Gu X Shen S Lin K Zhu C . Akr1d1 suppresses liver cancer progression by promoting bile acid metabolism-mediated nk cell cytotoxicity. Cell Metab. (2025) 37:1103–18 e7. doi: 10.1016/j.cmet.2025.01.011, PMID: 40010348

[190] Wang L Lankhorst L Bernards R . Exploiting senescence for the treatment of cancer. Nat Rev Cancer. (2022) 22:340–55. doi: 10.1038/s41568-022-00450-9, PMID: 35241831

[191] Shin E Bak SH Park T Kim JW Yoon SR Jung H . Understanding nk cell biology for harnessing nk cell therapies: targeting cancer and beyond. Front Immunol. (2023) 14:1192907. doi: 10.3389/fimmu.2023.1192907, PMID: 37539051 PMC10395517

[192] Pereira BI Devine OP Vukmanovic-Stejic M Chambers ES Subramanian P Patel N . Senescent cells evade immune clearance via hla-E-mediated nk and cd8(+) T cell inhibition. Nat Commun. (2019) 10:2387. doi: 10.1038/s41467-019-10335-5, PMID: 31160572 PMC6547655

[193] Ruggeri L Urbani E Andre P Mancusi A Tosti A Topini F . Effects of anti-nkg2a antibody administration on leukemia and normal hematopoietic cells. Haematologica. (2016) 101:626–33. doi: 10.3324/haematol.2015.135301, PMID: 26721894 PMC5004363

[194] Kim KM Noh JH Bodogai M Martindale JL Yang X Indig FE . Identification of senescent cell surface targetable protein dpp4. Genes Dev. (2017) 31:1529–34. doi: 10.1101/gad.302570.117, PMID: 28877934 PMC5630018

[195] Amor C Feucht J Leibold J Ho YJ Zhu C Alonso-Curbelo D . Senolytic car T cells reverse senescence-associated pathologies. Nature. (2020) 583:127–32. doi: 10.1038/s41586-020-2403-9, PMID: 32555459 PMC7583560

[196] Yang D Sun B Li S Wei W Liu X Cui X . Nkg2d-car T cells eliminate senescent cells in aged mice and nonhuman primates. Sci Transl Med. (2023) 15:eadd1951. doi: 10.1126/scitranslmed.add1951, PMID: 37585504

[197] Chaib S Lopez-Dominguez JA Lalinde-Gutierrez M Prats N Marin I Boix O . The efficacy of chemotherapy is limited by intratumoral senescent cells expressing pd-L2. Nat Cancer. (2024) 5:448–62. doi: 10.1038/s43018-023-00712-x, PMID: 38267628 PMC10965441

[198] Wang TW Johmura Y Suzuki N Omori S Migita T Yamaguchi K . Blocking pd-L1-pd-1 improves senescence surveillance and ageing phenotypes. Nature. (2022) 611:358–64. doi: 10.1038/s41586-022-05388-4, PMID: 36323784

[199] Borst L van der Burg SH van Hall T . The nkg2a-hla-E axis as a novel checkpoint in the tumor microenvironment. Clin Cancer Res. (2020) 26:5549–56. doi: 10.1158/1078-0432.CCR-19-2095, PMID: 32409305

[200] Chen M Jiang J Chen J Wang M Lu Y Liu L . The clinical safety and efficacy of targeted pd-L1 therapy with durvalumab in solid tumors. Curr Drug Targets. (2023) 24:584–98. doi: 10.2174/1389450124666230330101651, PMID: 36998143

[201] Buron M Etxebarria A Alvarez M Romayor I Eguizabal C . Natural killer cells in adoptive cell therapy: current landscape of genetic engineering strategies. Oncoimmunology. (2025) 14:2563099. doi: 10.1080/2162402X.2025.2563099, PMID: 40996824 PMC12477870

[202] Favaretto G Rossi MN Cuollo L Laffranchi M Cervelli M Soriani A . Neutrophil-activating secretome characterizes palbociclib-induced senescence of breast cancer cells. Cancer Immunol Immunother. (2024) 73:113. doi: 10.1007/s00262-024-03695-5, PMID: 38693312 PMC11063017

[203] McHugh D Gil J . Senescence and aging: causes, consequences, and therapeutic avenues. J Cell Biol. (2018) 217:65–77. doi: 10.1083/jcb.201708092, PMID: 29114066 PMC5748990

[204] Chien Y Scuoppo C Wang X Fang X Balgley B Bolden JE . Control of the senescence-associated secretory phenotype by nf-kappab promotes senescence and enhances chemosensitivity. Genes Dev. (2011) 25:2125–36. doi: 10.1101/gad.17276711, PMID: 21979375 PMC3205583

[205] Dou Z Ghosh K Vizioli MG Zhu J Sen P Wangensteen KJ . Cytoplasmic chromatin triggers inflammation in senescence and cancer. Nature. (2017) 550:402–6. doi: 10.1038/nature24050, PMID: 28976970 PMC5850938

[206] Gluck S Guey B Gulen MF Wolter K Kang TW Schmacke NA . Innate immune sensing of cytosolic chromatin fragments through cgas promotes senescence. Nat Cell Biol. (2017) 19:1061–70. doi: 10.1038/ncb3586, PMID: 28759028 PMC5826565

[207] Burton DGA Stolzing A . Cellular senescence: immunosurveillance and future immunotherapy. Ageing Res Rev. (2018) 43:17–25. doi: 10.1016/j.arr.2018.02.001, PMID: 29427795

[208] Kale A Sharma A Stolzing A Desprez PY Campisi J . Role of immune cells in the removal of deleterious senescent cells. Immun Ageing. (2020) 17:16. doi: 10.1186/s12979-020-00187-9, PMID: 32518575 PMC7271494

[209] Brighton PJ Maruyama Y Fishwick K Vrljicak P Tewary S Fujihara R . Clearance of senescent decidual cells by uterine natural killer cells in cycling human endometrium. Elife. (2017) 6:e31274. doi: 10.7554/eLife.31274, PMID: 29227245 PMC5724991

[210] Krizhanovsky V Yon M Dickins RA Hearn S Simon J Miething C . Senescence of activated stellate cells limits liver fibrosis. Cell. (2008) 134:657–67. doi: 10.1016/j.cell.2008.06.049, PMID: 18724938 PMC3073300

[211] Shi JW Lai ZZ Zhou WJ Yang HL Zhang T Sun JS . Tnfsf14(+) natural killer cells prevent spontaneous abortion by restricting leucine-mediated decidual stromal cell senescence. EMBO J. (2024) 43:5018–36. doi: 10.1038/s44318-024-00220-3, PMID: 39261664 PMC11535022

[212] Egashira M Hirota Y Shimizu-Hirota R Saito-Fujita T Haraguchi H Matsumoto L . F4/80+ Macrophages contribute to clearance of senescent cells in the mouse postpartum uterus. Endocrinology. (2017) 158:2344–53. doi: 10.1210/en.2016-1886, PMID: 28525591

[213] Munoz-Espin D Canamero M Maraver A Gomez-Lopez G Contreras J Murillo-Cuesta S . Programmed cell senescence during mammalian embryonic development. Cell. (2013) 155:1104–18. doi: 10.1016/j.cell.2013.10.019, PMID: 24238962

[214] Chen HA Ho YJ Mezzadra R Adrover JM Smolkin R Zhu C . Senescence rewires microenvironment sensing to facilitate antitumor immunity. Cancer Discov. (2023) 13:432–53. doi: 10.1158/2159-8290.CD-22-0528, PMID: 36302222 PMC9901536

[215] Kang TW Yevsa T Woller N Hoenicke L Wuestefeld T Dauch D . Senescence surveillance of pre-malignant hepatocytes limits liver cancer development. Nature. (2011) 479:547–51. doi: 10.1038/nature10599, PMID: 22080947

[216] Marin I Boix O Garcia-Garijo A Sirois I Caballe A Zarzuela E . Cellular senescence is immunogenic and promotes antitumor immunity. Cancer Discov. (2023) 13:410–31. doi: 10.1158/2159-8290.CD-22-0523, PMID: 36302218 PMC7614152

[217] Vivier E Raulet DH Moretta A Caligiuri MA Zitvogel L Lanier LL . Innate or adaptive immunity? The example of natural killer cells. Science. (2011) 331:44–9. doi: 10.1126/science.1198687, PMID: 21212348 PMC3089969

[218] Maia A Tarannum M Lerias JR Piccinelli S Borrego LM Maeurer M . Building a better defense: expanding and improving natural killer cells for adoptive cell therapy. Cells. (2024) 13:451. doi: 10.3390/cells13050451, PMID: 38474415 PMC10930942

[219] Santoni A Zingoni A Cerboni C Gismondi A . Natural killer (Nk) cells from killers to regulators: distinct features between peripheral blood and decidual nk cells. Am J Reprod Immunol. (2007) 58:280–8. doi: 10.1111/j.1600-0897.2007.00513.x, PMID: 17681044

